# DECTNet: Dual Encoder Network combined convolution and Transformer architecture for medical image segmentation

**DOI:** 10.1371/journal.pone.0301019

**Published:** 2024-04-04

**Authors:** Boliang Li, Yaming Xu, Yan Wang, Bo Zhang

**Affiliations:** 1 Department of Control Science and Engineering, Harbin Institute of Technology, Harbin, Heilongjiang, China; 2 Sergeant Schools of Army Academy of Armored Forces, Changchun, Jilin, China; Vellore Institute of Technology: VIT University, INDIA

## Abstract

Automatic and accurate segmentation of medical images plays an essential role in disease diagnosis and treatment planning. Convolution neural networks have achieved remarkable results in medical image segmentation in the past decade. Meanwhile, deep learning models based on Transformer architecture also succeeded tremendously in this domain. However, due to the ambiguity of the medical image boundary and the high complexity of physical organization structures, implementing effective structure extraction and accurate segmentation remains a problem requiring a solution. In this paper, we propose a novel Dual Encoder Network named DECTNet to alleviate this problem. Specifically, the DECTNet embraces four components, which are a convolution-based encoder, a Transformer-based encoder, a feature fusion decoder, and a deep supervision module. The convolutional structure encoder can extract fine spatial contextual details in images. Meanwhile, the Transformer structure encoder is designed using a hierarchical Swin Transformer architecture to model global contextual information. The novel feature fusion decoder integrates the multi-scale representation from two encoders and selects features that focus on segmentation tasks by channel attention mechanism. Further, a deep supervision module is used to accelerate the convergence of the proposed method. Extensive experiments demonstrate that, compared to the other seven models, the proposed method achieves state-of-the-art results on four segmentation tasks: skin lesion segmentation, polyp segmentation, Covid-19 lesion segmentation, and MRI cardiac segmentation.

## Introduction

In order to assist physicians in understanding and analyzing medical images, medical image segmentation has received increasing attention due to its efficient and effective characteristics in recent years [1], such as skin lesion segmentation [2–4], cardiac and myocardial segmentation [5–8], colonoscopic polyp segmentation [9–12], and Covid-19 lesion segmentation [13–15]. Accurate semantic segmentation of medical images significantly facilitates quantitative pathology assessment, treatment planning, and disease progression monitoring. However, manual image segmentation is time-consuming, requiring expertise and limited reproducibility. Therefore, it is essential to develop approaches to achieve automatic medical image segmentation.

Convolution Neural networks(CNNs) have achieved considerable success in medical image analysis in the past decade. They have achieved state-of-the-art performance in a large number of segmentation tasks. Compared with traditional methods, CNNs have superior modeling representation capability and automatically learn task-relevant features in medical images. In particular, the most extensive application is the UNet [16] architecture based on the encoder and decoder. In UNet, the encoder analyzes the semantic image information and learns high-dimensional features, and the decoder performs image segmentation according to the representations. Since the encoder lacks the details of the original image when learning high-dimensional representations, skip connections are employed to supplement the detailed information in the decoder’s features, and this technique significantly improves the image segmentation performance.

With the great success of Transformer architecture in the field of NLP, several studies have been devoted to transferring Transformer architecture to the computer vision domain. The Vision Transformer(ViT) model [17] is the first to demonstrate the efficacy of the Transformer architecture in computer vision. The advantage of the ViT model is that it can establish the dependence between long-term pixels in images, improving the performance in visual tasks. Swin Transformer [18] also demonstrates the efficacy of Transformer architecture with hierarchical representations for vision tasks. However, different from CNNs, the Transformer architectures need more annotated samples to perform better than the convolutional models due to the lack of inductive bias. The convolution-based models can extract low-dimensional semantic information from images and establish fine spatial detail features, but they are not perfect for establishing global relationships due to the limits of the receptive field. On the contrary, the Transformer architectures provide excellent compensation by achieving global information modeling, which is the drawback of the convolution-based models. For this reason, we combine the advantages of convolution and Transformer structures to design a novel medical image segmentation model.

Specifically, we propose a segmentation model with a dual encoder that enables combining the advantages of the convolution and Transformer architectures. The convolution encoder with channel and spatial attention can sufficiently extract the local context information. In contrast, the Swin Transformer architecture is utilized as the fundamental component of the Transformer encoder, which is based on the window and shift-window self-attention technique and is sensitive to global context information. It is proved in [19, 20] that the visual features extracted by the Transformer architecture differ from the convolution architecture. Guo *et al*. [21] proves that a strong backbone is a key to semantic segmentation. In addition, to improve the Swin Transformer architecture for dense prediction tasks, we designed a STP Block in the Swin Transformer encoder to enable the encoder to extract shallow semantic information in the initial stage.

Similar to UNet, DECTNet employs the skip connection between the encoder and the decoder. However, because the stage of the convolutional encoder outputs features at different scales from the stage of the Transformer encoder, we design a feature fusion decoder stage. It can fuse features of different scales from different stages and select the representations beneficial to specific tasks by the unique feature selection module. We further design a deep supervision module to supervise the decoder stage outputs. It can accelerate the convergence of the model and enable the model to obtain superior segmentation performance.

Like our proposed DECTNet, Li *et al* [22] designed a segmentation model with a dual encoder based on convolution and Transformer architectures named CATS. However, CATS applies the vanilla ViT [17] architecture in the Transformer-based encoder, which increases the parameters and the computational complexity of the segmentation model. In addition, the DECTNet contains abundant channels and spatial attention, feature selection, and fusion modules to extract suitable representations from redundant features for specific segmentation tasks, which is not available with the CATS method but is essential for dense prediction tasks. In summary, the main contributions of this paper are reflected in the following three aspects:

- We propose a novel segmentation method called DECTNet, which has two encoders based on convolution and Swin Transformer architectures. The convolution-based encoder through the dense connections and CBAM module enables to select the specific task-relevant local features. Meanwhile, by combining the STB module, the standalone and entire Swin Transformer encoder allows the efficient establishment of the global representation in images.
- To learn knowledge of the features from different encoders, we design a practical feature fusion decoder, which integrates the representation from the convolution encoder and the Swin Transformer encoder. It enables to select the valuable information from features for segmentation tasks.
- We perform extensive experiments in four medical image segmentation tasks. Experiments show that our model performs best on most metrics compared to other state-of-the-art models. Furthermore, the ablation experiments demonstrated the effect of each component of the proposed DECTNet.

The remainder of this paper is arranged as follows. Section **Related works** introduces the related works. Section **Materials and methods** describes the proposed DECTNet in detail. Section **Experiment** and Section **Results** present the experiment implementation and results. Section **Discussion** and Section **Conclusion** contain the discussion and conclusions.

## Related works

According to the different network structures, the existing medical image segmentation methods based on deep learning can be divided into two categories: 1) the methods based on convolution structures; and 2) the methods that combine convolution and the self-attention mechanism. Although some models entirely adopt the self-attention mechanism as the feature extraction technology, the number of these models is relatively small, so we classify them into the second category.

### Methods based on convolution network

In recent years, numerous convolution-based segmentation models have significantly progressed in various segmentation tasks. In particular, FCN [23] is one of the most notable approaches. It has the analysis and synthesis path consisting of cascaded convolution, pooling, and deconvolution layers. Compared to FCN, the UNet has a skip connection between the analysis and synthesis paths, which supplements the detailed information lacking in the features of synthesis paths. Because of its flexible structure, UNet is widely applied in medical image segmentation.

Compared to UNet, the UNet++ [24] and CPFNet [25] apply additional skip connections between the encoder and decoder to aggregate features from different semantic scales. In addition, DeepLabv3+ [26] and CENet [27] employ multiple convolutional branches with various receptive fields to improve the multi-scale information capture capability of the model.

As the channel and spatial attention are effective in visual tasks [28, 29], several approaches combining convolution and attention mechanism have emerged. For example, [30, 31] use channel attention to guide shallow-level learning of global feature representation. In contrast, the attention UNet proposed in [32] combines spatial attention with UNet structure for abdominal pancreas segmentation from CT images. Roy *et al*. [33] propose a scSE framework that combines spatial attention and channel attention, which has been proven effective on whole brain and abdominal multiple organs segmentation tasks. In addition, Song *et al*. [34] and other works [35, 36] also start from the perspective of the combination of convolution and attention mechanism to design models to deal with specific medical image segmentation tasks.

### Methods combined convolution with self-attention mechanism

With the success of the Transformer architecture in natural language processing, its application to computer vision has become a popular research domain. ViT [17] is the first method to successfully employ the Transformer architecture to solve computer vision problems. It divides images into patches and establishes global context dependencies by computing the similarity between patches. The Swin Transformer [18] is an improved version of the ViT. It introduces the window and shift-window self-attention module to reduce the computational complexity of the Transformer structures. Furthermore, different from ViT, Swin Transformer allows for producing multiple scales of features, essential for applying the Transformer structure on dense predictions, such as object detection and image segmentation.

Recently, researchers have attempted to combine traditional convolution with the self-attention mechanism to design models with superior performance in medical image segmentation tasks. In particular, TransUNet [37] combines ViT and UNet, which employs an encoder-decoder and convolution portion to extract local contextual information in high-resolution, low-dimension features. It also uses the Transformer portion to establish the global relationships of patches in low-resolution, high-dimension features. TransUNet establishes global dependencies between patches of high-dimensional representation through self-attention but fails to establish long-term reliance between the patches of low-dimensional semantic features. Cao *et al*. [38] proposed the SwinUNet, which employs the Swin Transformer Blocks in the UNet backbone instead of convolution as the feature extraction technique. After the pre-training process, this method performs excellently on multi-organ and cardiac segmentation tasks.

In addition, Hung *et al*. [39] designed a cross-slice attention Transformer module. Combining it with convolutional networks and skip connections enables the accurate segmentation of prostate partitions in MRI. Liu *et al*. [40] designed the MCTHNet by integrating convolution and transformer structures for multi-modal medical image segmentation with limited annotation, and their approach achieved the best semi-supervised results on several multi-modal datasets. Furthermore, TransFuse [41], Medical Transformer [42], TransBTS [43], FCT [44], and HiFormer [45] combine self-attention with convolutional networks to achieve excellent results in specific medical image segmentation tasks.

Although increasing research has been focused on combining the self-attention mechanism and convolution to improve the performance of segmentation models, only some studies have noticed that the strong backbone of the Transformer architecture may have positive impacts on image segmentation. Therefore, we combine the Transformer structure rather than the simple self-attention technique with convolution architecture.

## Materials and methods

### Overview

**Fig 1:**
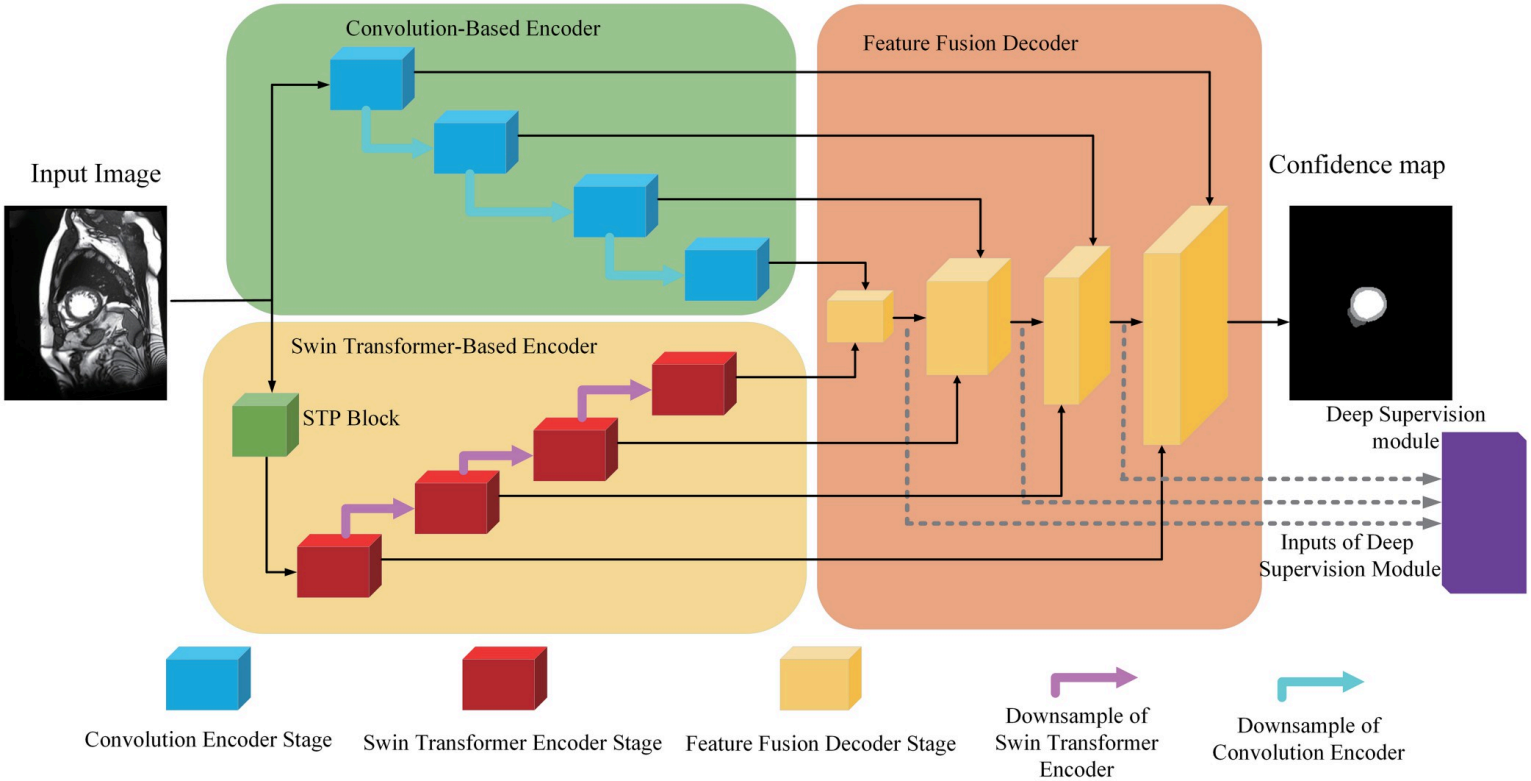
Overview of our proposed DECTNet approach with dual-encoder-single-decoder structure. DECTNet consists of four components: Convolution-based encoder, Swin Transformer-based encoder, Feature Fusion decoder, and Deep Supervision module. The detailed composition of each component is described in the following sections.

Fig 1 illustrates the overall architecture of our proposed DECTNet. The DECTNet is based on two-encoders-single-decoder architecture and consists of four main parts: convolution structure encoder, Swin Transformer structure encoder, feature fusion decoder, and deep supervision module. For the convenience of expression, we denote the four main components as C-Encoder, ST-Encoder, F-Decoder, and DS-Module, respectively. In the following subsections of this section, we describe the structure of each component of the proposed method in detail.

### Convolution-based encoder

**Fig 2:**
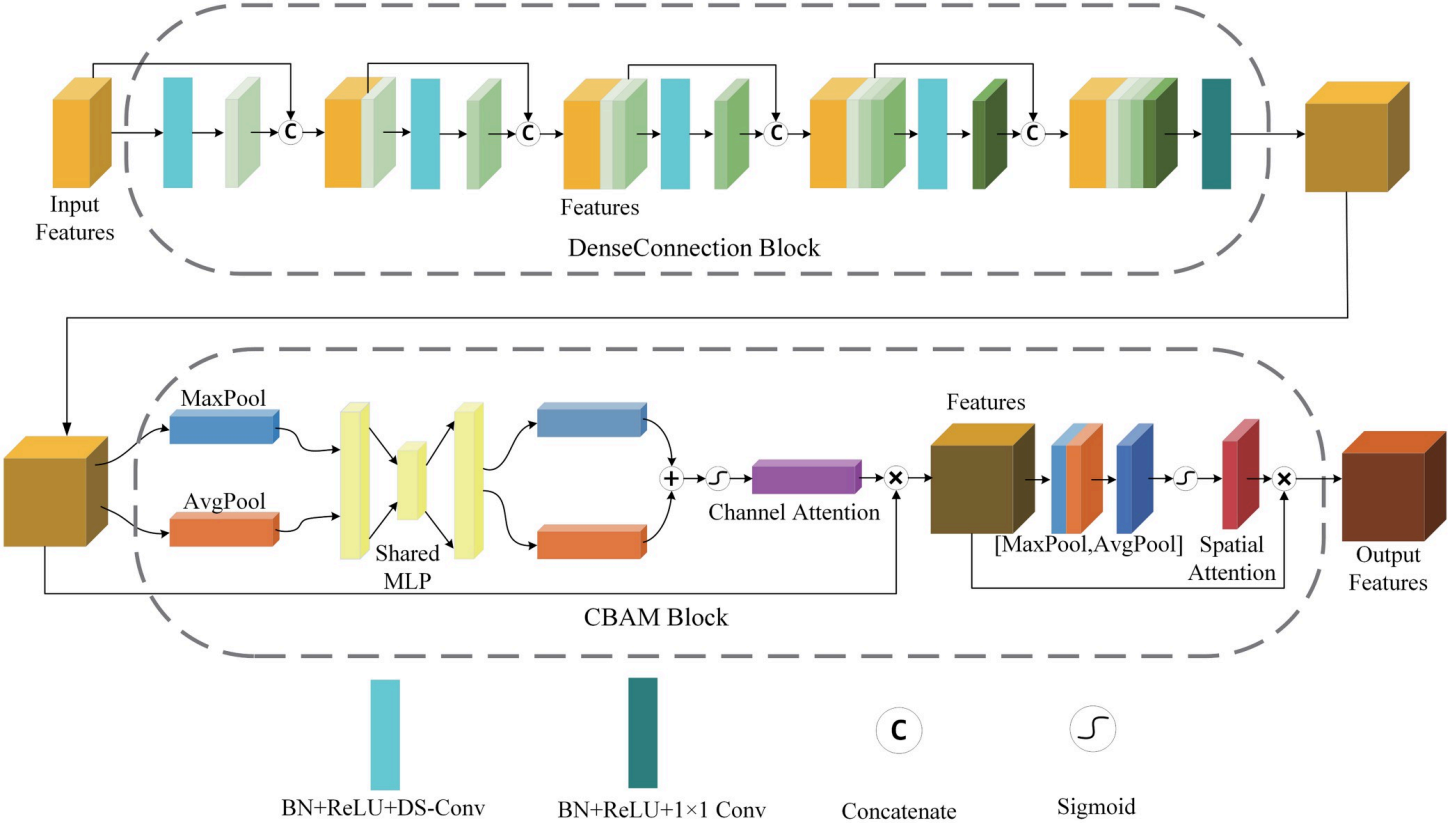
The detailed structure of the convolution encoder stage. This stage consists of the DenseConnection Block and the CBAM Block, which are applied to sufficiently extract detailed information from the images.

As shown in Fig 1, the convolution-based encoder of the DECTNet consists of four stages. Like the UNet encoder stage, each part in the C-encoder has the same structure but operates on different scale features. Except for the deepest stage, there is a downsampling operation between different stages, which reduces the scale of the features but increases the channels of features to extract high-dimension representations. The detailed design of the single stages is shown in Fig 2.

Fig 2 demonstrates the detailed structure of the C-encoder stage. Each stage combines a dense connection block [46] and a CBAM block [28]. In a single stage, the features pass into the dense connection block for adequate information extraction and then through the CBAM module to further enhance the valuable information for the segmentation task. It should be noted that in a single stage, the scale and channels of the features are not changed.

Compared with the Residual block, the dense connection block has a more abundant residual connection, which can fully utilize features and reduce the impact of gradient disappearance. When the features are output from the dense connection block, they are fed to the CBAM module. The role of the CBAM module is to add a spatial attention mask and channel attention mask to the features. It can make the model pay more attention to the task-related information and ignore the task-independent information of the features. Compared to the SE module [29], the CBAM module adds more affluent spatial attention, enhancing the valid information in the features.

Due to the DECTNet having two encoders, the model parameters increase significantly. In order to reduce the model parameters, we use the depth separable convolution [47] instead of the traditional convolution in the C-encoder. In addition, we use a traditional convolution with a step length of 2 and a convolution kernel size of 2 as the downsampling operation, reducing the scale of the features while increasing its channels.

### Swin Transformer-based encoder

**Fig 3:**
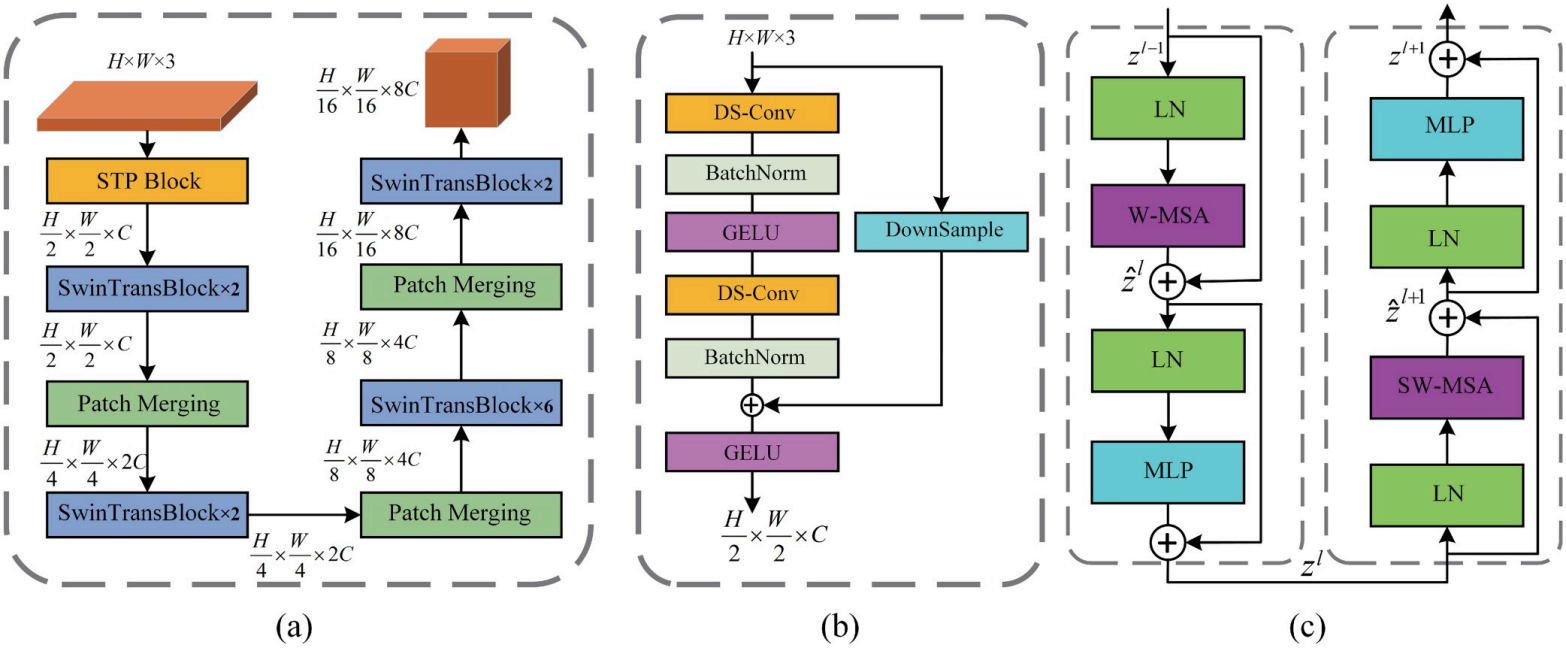
Overview of the Swin Transformer-based encoder of the proposed DECTNet. (a) Components of the SwinTransformer-based encoder. It includes scaling the features in the ST-Encoder. (b) Composition of the STP block (c) Two successive Swin Transformer Block. W-MSA and SW-MSA are multi-head self-attention modules with regular and shifted windowing configurations.

The other encoder of the DECTNet consists of the Swin Transformer architecture, detailed in Fig 3(a). Compared with the original ViT architecture, the Swin Transformer structure yields hierarchical features essential for image segmentation. Fig 3(a) also shows the scale of features through each stage of the ST-Encoder. The structure of Patch Merging blocks is identified with the Swin Transformer [18].

Compared with image recognition, semantic segmentation needs more semantic details and structured information, such as spatial context information. Therefore, using large steps to initialize the image, just as in the traditional ViT method, results in the absence of semantic information in raw images. Small-step convolution operation instead of this process improves the performance of models for semantic segmentation tasks. Therefore, we designed the STP block, as shown in Fig 3(b). It extracts low-dimensional features from the image using the structure of small-step convolution of residual blocks. The purpose of the STP block is to adjust the resolution and channels of low-dimensional features to provide features rich in detailed semantic information to ST-Encoder stages, thereby improving the segmentation performance of the method.

The structure of the Swin Transformer Block is shown in Fig 3(c). Compared with the traditional ViT architecture, It utilizes a window-based self-attention module (W-MSA) and a shifted window-based self-attention module (SW-MAS) to improve computational efficiency. The W-MSA and the SW-MSA structures are described in [18]. In the Swin Transformer Block, the procedure for processing the features can be formulated as follows:

$$\begin{array}{c} {\hat{z}}^{1}=\text{W-MSA}(\text{LN}(z^{1-1}))+z^{1-1} \end{array}$$ (1)

$$\begin{array}{c} z^{1}=\text{MLP}(\text{LN}({\hat{z}}^{1}))+{\hat{z}}^{1} \end{array}$$ (2)

$$\begin{array}{c} {\hat{z}}^{1+1}=\text{SW-MSA}(\text{LN}(z^{1}))+z^{1} \end{array}$$ (3)

$$\begin{array}{c} z^{1+1}=\text{MLP}(\text{LN}({\hat{z}}^{1+1}))+{\hat{z}}^{1+1} \end{array}$$ (4)

where ${\hat{z}}^{l}$ and *z*^*l*^ are the output features of the (S)W-MSA and the MLP module at layer *l*. Due to the complementary and similarities between the W-MAS and the SW-MSA, even numbers of Swin Transformer Block are required at each Swin Transformer encoder stage, which is [2, 2, 6, 2] in the proposed DECTNet, just as shown in Fig 3(a).

### Feature fusion decoder

**Fig 4:**
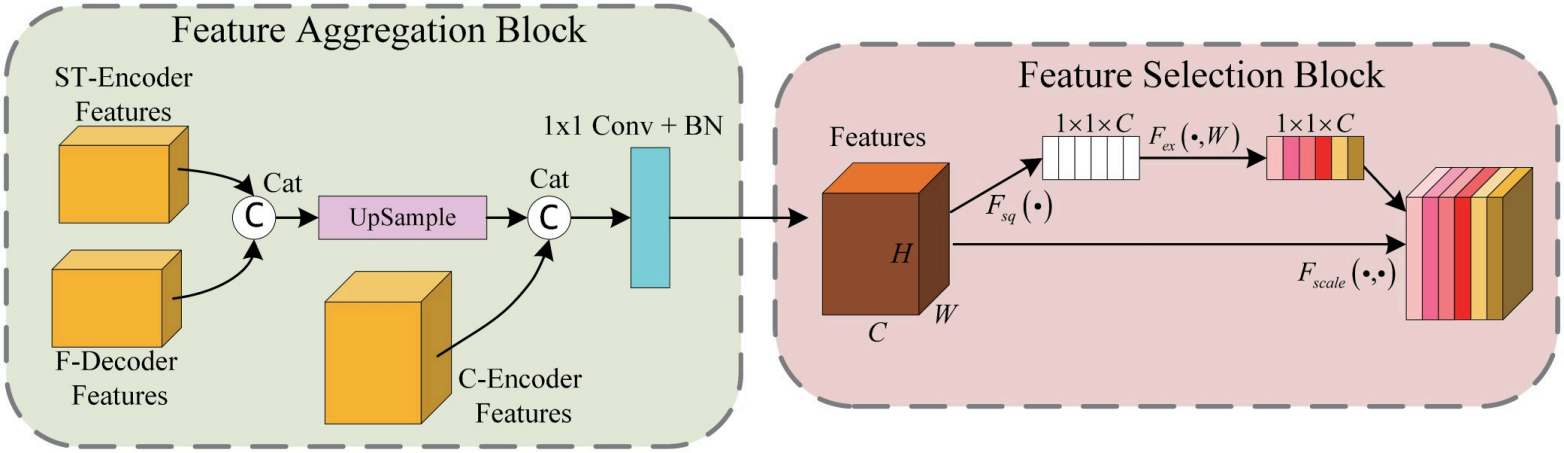
The detailed structure of the feature fusion decoder stage. The stage has two components: the feature aggregation block and the feature selection block, which are applied to integrate and select features.

Due to the dual encoder architecture of the DECTNet, the decoder is required to receive different scale features from the skip connection. Therefore, we design a novel Feature Fusion Decoder to suit the particular structure of the proposed method. As shown in Fig 1, similar to the two encoders in the DECTNet, the proposed F-Decoder consists of several same structure stages. In addition to the stage that processes the lowest resolution features, each decoder stage receives three collections of features from the skip connections of the two encoder stages and the prior stage in the F-decoder. Fig 4 illustrates the details of the composition of a F-Decoder stage.

Fig 4 clearly shows that the stage of the F-decoder consists of a feature aggregation block and a feature selection block. The role of the feature aggregation block is to unify the different resolution features from different parts and adjust the redundant channels of the features. It first connects the features from the ST-Encoder stage and the prior stage of the F-Decoder. Then, it adjusts the channels and resolution by transposed convolution for the two combined features and concatenates the features from the C-encoder stage with the combined features. Finally, it adjusts the channels of the features by 1×1 convolution and batch normalization operations to suit the feature selection block.

The purpose of the feature selection block is to reinforce the meaningful information about the features from the feature aggregation block through the attention mechanism. It applies the SE module [29] to add channel attention to the features, enhancing features that benefit tasks and ignoring the tasks-irrelevant features. It should be noted that the initial stage of the F-Decoder, that is, the intersection of the C-Encoder stage and the ST-Encoder stage, has only two part features as inputs, which are from the C-Encoder stage and the ST-Encoder stage since there are no features from the prior stage of F-Decoder.

### Deep supervision and loss function

**Fig 5:**
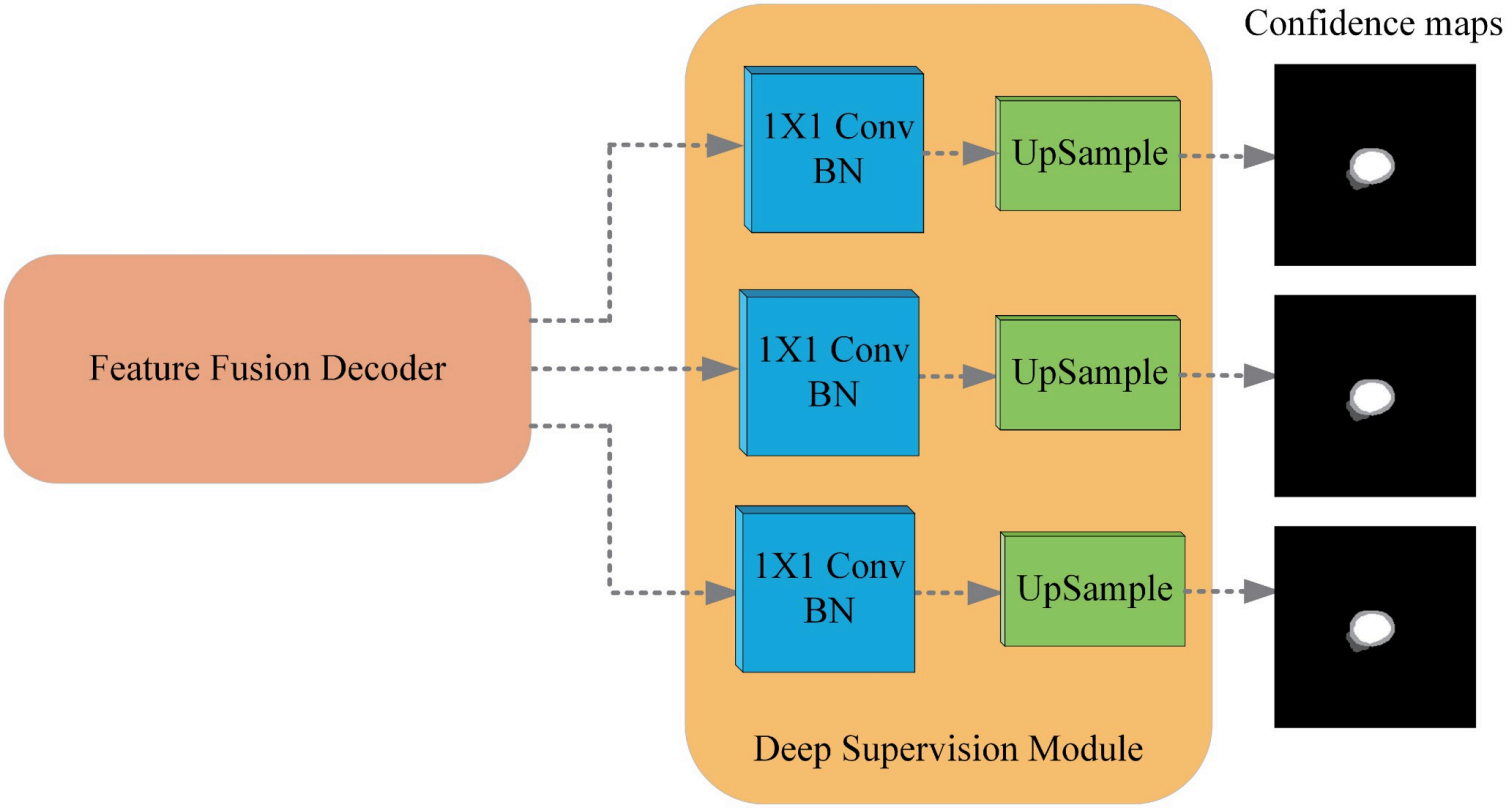
The detailed structure of the deep supervision module. The DS-Module converts different scale features of the F-decoder into the same scale confidence maps.

It demonstrates in [48, 49] that the deep supervision technique could accelerate the convergence of the segmentation method and achieve better performance. For this reason, we introduce a deep supervision module to supervise the output features of decoder stages. The detail of the DS-Module is shown in Fig 5.

The structure of the DS-Module is quite simple. For the output features of a particular decoder stage, the DS-Module consists of the Convolution, Batch Normalization, and UpSample operations, where transposed convolution is adopted to implement UpSample. For features of different scales output from different stages, DS-Module first uses 1 × 1 convolution and batch normalization to adjust the number of channels to the same. Then it uses transpose convolution to upsample feature size from different stages to label size, and the channels are adjusted to classification quantity. Thus, the confidence maps produced by the F-Decoder and DS-Module have identical resolutions.

Both the F-Decoder and the DS-Module output confidence maps with the same resolution, and each confidence map generates a loss with the label during the training process. Therefore, the loss function of the DECTNet can be defined as the sum of all losses between the prediction confidence maps and the corresponding labels. It can be defined as:

$$\begin{array}{c} L_{total}=\sum_{k=1}^{K}\omega^{k}l^{k} \end{array}$$ (5)

where *K* represents the quantity of prediction confidence maps, *l*^*k*^ represents the loss calculated by the *k*th prediction confidence map and segmentation mask, and *ω*^*k*^ is the weight of the corresponding loss. In this paper, we apply Dice Loss and cross-entropy loss as the *l*^*k*^, which can be defined as:

$$\begin{array}{c} l^{\left(k\right)}=\alpha \cdot l_{ce}^{\left(k\right)}+\left(1-\alpha \right)\cdot l_{Dice}^{\left(k\right)} \end{array}$$ (6)

In DECTNet, *K* and *α* are set to 4 and 0.5, respectively. *l*^4^ refers to the loss resulting from the final confidence maps of the model against the ground truth. *l*^1^-*l*^3^ are the losses established from the confidence maps output by the DS-Module. To ensure the dominance of *l*^4^ in overall loss, we set *ω*^4^ as 0.7, and *ω*^1^-*ω*^3^ are all set to 0.1.

## Experiment

### Datasets

In this paper, we evaluate the segmentation performance of the proposed DECTNet in four medical image segmentation tasks: skin lesion segmentation, Covid-19 lesion segmentation, polyp segmentation, and cardiac segmentation. For the skin lesion segmentation task, we utilize the ISIC2017 dataset [50], collected from different leading clinical centers internationally and acquired from different devices. This dataset includes 2000 images for training, 150 for validation, and 600 for testing.

The QaTa-COV19 dataset [51] is employed for the Covid-19 lesion segmentation task, collected by researchers from Qatar University and Tampere University. This dataset contains 9258 chest radiographs of COVID-19 lesions with manual annotation, which includes 7145 images in the training dataset and 2113 images in the test dataset. And we choose randomly 750 samples from the training dataset as the validation dataset.

Following [41], we adopt five public datasets in the polyp segmentation task: Kvasir [52], CVC-ClinicDB [53], CVC-ColonDB [54], EndoScene [55], and ETIS [56]. The same split as described in [57, 58] are adopted, i.e., 1450 training images are solely selected from Kvasir and CVC-ClinicDB while 798 testing images are from all five datasets. In addition, we randomly choose 10% of the training samples as the validation dataset.

The dataset for the cardiac segmentation task is provided by the Multi-Centre, Multi-Vendor & Multi-Disease (M&Ms) Cardiac Image Segmentation Challenge [59], which was acquired at six different clinical centers using MRI scanners from four vendors. The samples are segmented by experienced clinicians from the respective institutions, including contours for the left ventricle(LV) and right ventricle (RV), as well as for the left ventricular myocardium (MYO). Due to the M&Ms dataset containing 3D samples, we convert the 3D voxel samples into 2D slices, in which the training dataset, validation dataset, and test dataset, respectively, consist of 3518, 450, and 1024 images.

### Implementing details and evaluation metrics

The implementation of the proposed DECTNet is based on the public PyTorch platform and NVIDIA GeForce RTX 3090. During the training process, we adopt stochastic gradient descent(SGD) as the optimizer to optimize our method, where the momentum and weight decay are set to 0.9 and 0.0001, respectively. The initial learning rate is set to 0.004, and the “poly” learning rate policy is adopted, which is the initial learning rate multiplied by ${\left(1-\frac{iter}{total\_iter}\right)}^{power}$, where power is set to 0.9. In addition, The batch size and maximum epoch are set to 16 and 100, respectively.

Since samples from different datasets have different scales and distributions, firstly, we uniformly resized the input samples as 224×224. Then we normalized the samples as zero mean and unit variance, and finally, the random rotation and flip operations were adopted as the data augmentation strategy. In DECTNet, the total number of training parameters is 12.6M, with the initial channels of the convolution structure set to 32 and the initial channels of the transformer structure set to 48. When the initial channels of the convolution structure are set to 16 and the initial channels of the transformer structure are set to 24, the total number of training parameters is reduced to 3.2M.

In order to adequately evaluate the performance of the proposed method, we employ eight evaluation metrics in the four segmentation tasks, which are the Dice similarity coefficient(DSC), Jaccard Index(Jacc), Accuracy(Accu), Sensitivity(Sens), Precision(Prec), Specificity(Spec), Average symmetric surface distance(ASD) and Hausdorff distance (HD). The different evaluation metrics are utilized for different segmentation tasks.

In addition, we compared the proposed DECTNet with seven other segmentation methods, which are UNet [16], UNet++ [24], AttentionUNet [32], DeepLabV3+ [26], CENet [27], TransUNet [37], TransFuse [41]. For a fair comparison, all the segmentation models are trained from scratch.

## Results

### Skin lesion segmentation

We first verify the effect of the proposed DECTNet in the skin lesion segmentation task. The Dice, Jaccard, Accuracy, Sensitivity, Precision, and Specificity are adopted as the evaluation metrics in this task. Table 1 shows the quantitative results of our model and other segmentation methods. Our model achieved the best performance in mean Dice, Jaccard, Accuracy, and Sensitivity, which are 86.36%, 78.38%, 94.91%, and 84.46%, respectively. Compared to the best results produced by other methods, the results obtained from DECTNet are enhanced by 0.70% on Dice(85.66%, CENet), 0.96% on Jaccard(77.42%, CENet), 0.81% on Accuracy(94.10%, CENet) and 0.63% on Sensitivity(83.83%, Transfuse). Although our method does not achieve the best results in Precision and Specificity, it remains competitive compared to other methods.

**Table 1:** Result of comparisons with other methods in skin lesion segmentation task.

	Metrics					
Method	Dice(%)	Jaccard(%)	Accuracy(%)	Sensitivity(%)	Precision(%)	Specificity(%)
UNet	82.58(0.51)	73.40(0.61)	92.32(0.05)	81.33(0.30)	90.31(0.18)	96.44(0.19)
UNet++	83.22(0.14)	74.22(0.33)	92.70(0.15)	81.39(0.64)	91.70(0.34)	97.38(0.23)
AttnUNet	82.94(0.67)	73.33(0.46)	92.40(0.25)	81.14(0.49)	90.19(0.21)	97.43(0.21)
DeepLabV3+	83.06(0.47)	74.03(0.30)	92.71(0.21)	81.89(0.29)	90.92(0.50)	97.59(0.21)
CENet	85.66(0.31)	77.42(0.54)	94.10(0.21)	82.72(0.35)	91.97(0.41)	**97.92**(0.20)
TransUNet	84.50(0.56)	76.28(0.62)	93.02(0.14)	82.42(0.55)	**92.97**(0.24)	97.41(0.21)
TransFuse	85.22(0.27)	76.90(0.54)	93.67(0.11)	83.83(0.30)	92.01(0.33)	96.98(0.09)
DECTNet	**86.36**(0.38)	**78.38**(0.49)	**94.91**(0.13)	**84.46**(0.31)	92.40(0.36)	97.12(0.12)

The values are described as Mean(Standard deviation). Best results are in bold and suboptimal results are in underlined.

**Fig 6:**
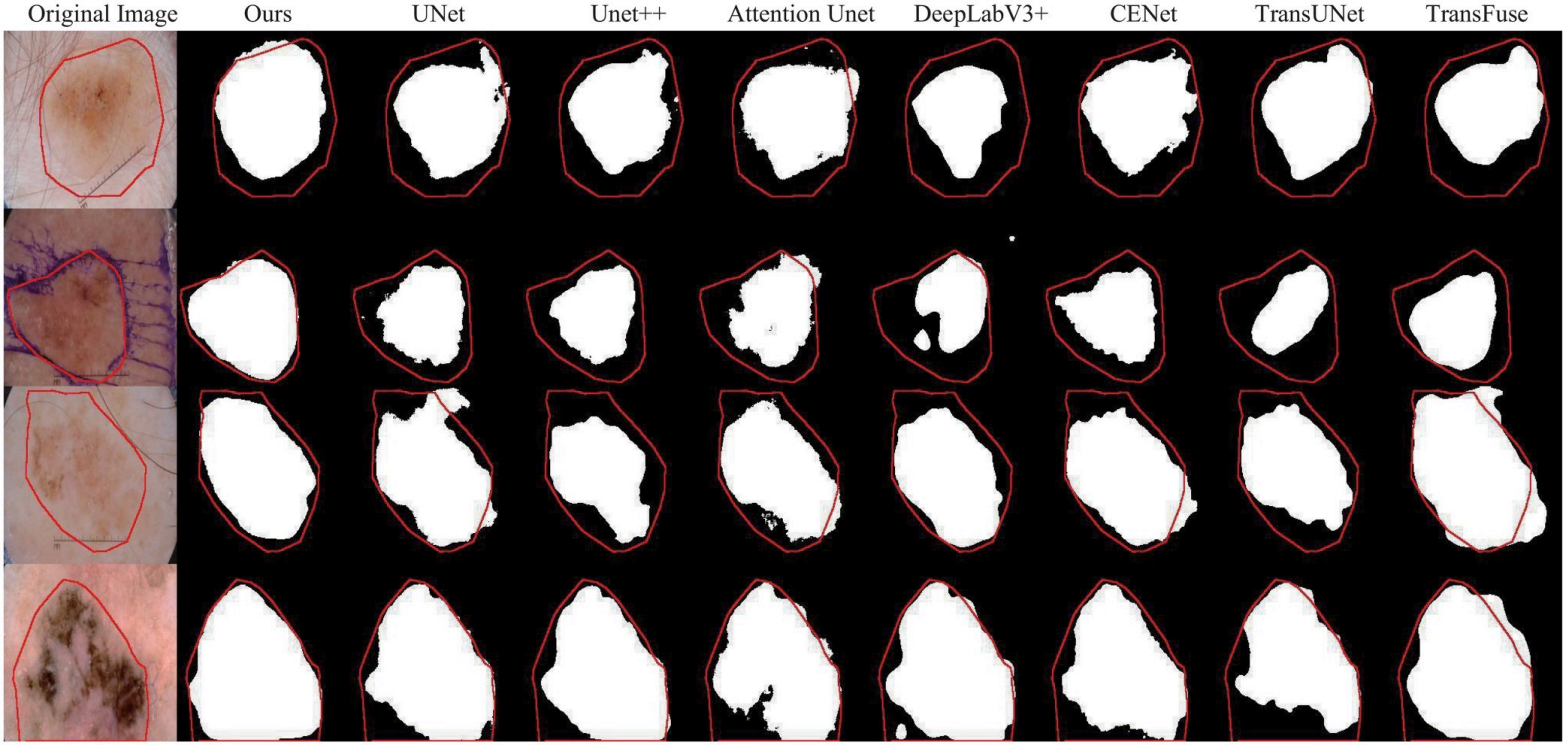
Visual comparison examples with other approaches in the skin lesion segmentation. The red contour refers to the ground truth, and different segmentation masks are produced by different methods.

Fig 6 gives several visual samples and segmentation masks produced by our and other methods. The red curves in the figure are the contours of the ground truth corresponding to the samples. For convenient contrast, we integrate ground truth contours with the samples and the segmentation masks derived from the different methods. These samples show that the segmentation masks of our approach are very close to the ground truth. In contrast with the segmentation masks of other methods, our masks are a better fit for the lesions that need to be selected.

**Fig 7:**
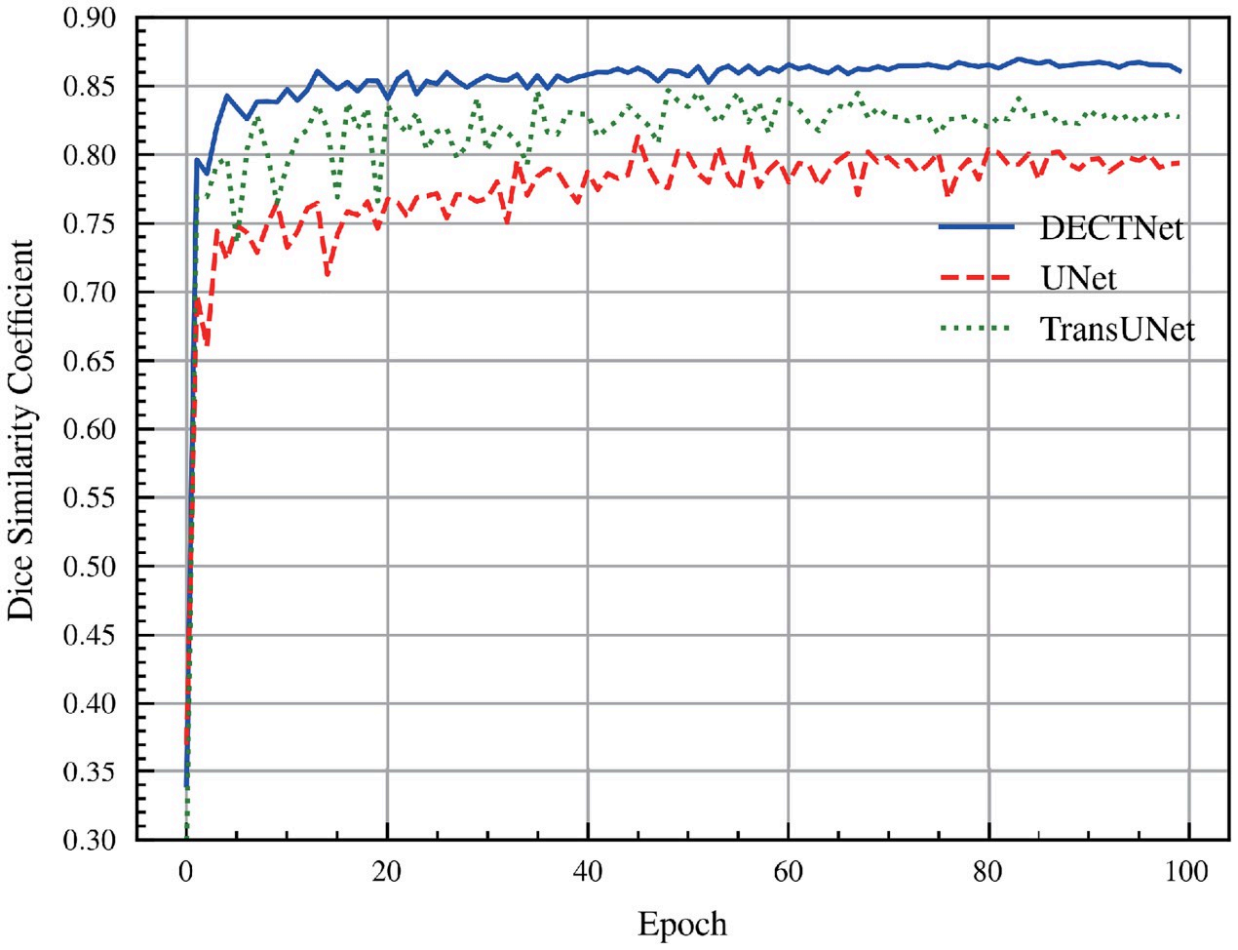
Comparison of Dice score among different methods on the validation dataset in the skin lesion segmentation task during the training process.

Fig 7 depicts the Dice score of different approaches on the validation dataset during the training process of the skin lesion segmentation task. For the convenience of observation, the figure merely depicts the Dice score curves on the validation set for three segmentation models, which are UNet, TransUNet, and DECTNet, respectively. It is shown that the curve acquired by our method is smoother and achieves a higher Dice score on the validation dataset than UNet and TransUNet.

### Covid-19 lesion segmentation

**Fig 8:**
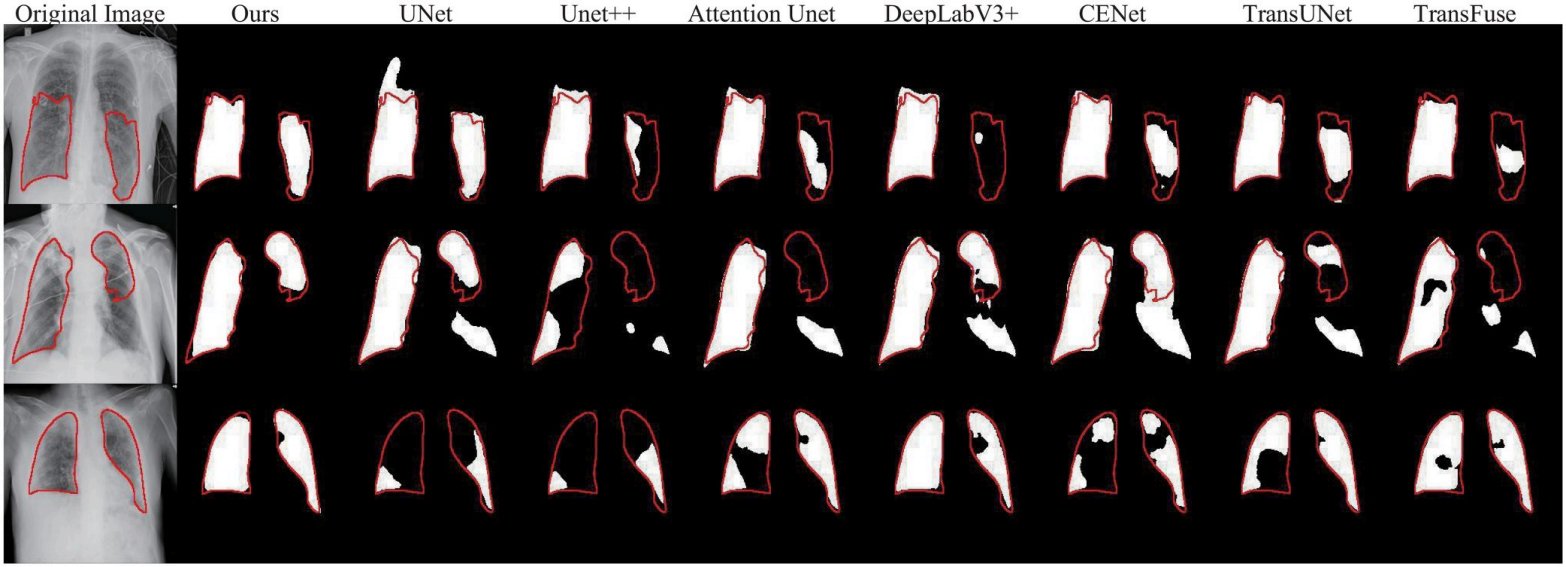
Visual comparison examples with other approaches in the Covid-19 lesion segmentation. The red contour refers to the ground truth, and different segmentation masks are produced by different methods.

Then, we evaluate the performance of the proposed DECTNet in the Covid-19 lesion segmentation task. The comparison experiment for the Covid-19 segmentation task is similar to the skin lesion segmentation task. Table 2 and Fig 8 respectively indicate the corresponding quantified results and visual comparisons of our model and seven other segmentation methods on the Covid-19 lesion dataset. Similar to the results on the skin lesion dataset, our DECTNet is also able to obtain the best performance in terms of the mean Dice, Jaccard, Accuracy, and Precision metrics in the Covid-19 dataset. Compared to UNet in Table 2, our approach achieves significant gains in each evaluation metric, which intuitively demonstrates the effectiveness of the diverse modules designed in DECTNet.

**Table 2:** Result of comparisons with other methods in Covid-19 lesion segmentation task.

	Metrics					
Method	Dice(%)	Jaccard(%)	Accuracy(%)	Sensitivity(%)	Precision(%)	Specificity(%)
UNet	83.87(0.31)	72.92(0.67)	96.58(0.45)	87.04(0.86)	81.31(0.89)	97.65(0.12)
UNet++	83.74(1.02)	72.71(0.81)	96.61(0.37)	85.58(0.75)	82.54(0.61)	97.87(0.55)
AttnUNet	83.68(0.67)	72.63(0.61)	96.53(0.25)	87.06(0.40)	81.03(0.52)	97.66(0.24)
DeepLabV3+	84.43(0.85)	73.67(0.46)	96.75(0.21)	86.36(0.45)	82.95(0.95)	97.92(0.20)
CENet	84.62(0.73)	74.03(0.62)	96.75(0.45)	88.01(0.39)	81.90(0.66)	97.71(0.11)
TransUNet	85.49(1.02)	75.26(0.82)	96.96(0.50)	87.04(0.76)	84.33(0.44)	98.13(0.18)
TransFuse	85.18(0.85)	74.79(0.46)	96.80(0.17)	**89.77**(0.33)	85.54(0.72)	**98.62**(0.17)
DECTNet	**87.99**(0.37)	**78.96**(0.21)	**97.50**(0.46)	89.00(0.50)	**87.27**(0.63)	98.51(0.12)

The values are described as Mean(Standard deviation). Best results are in bold and suboptimal results are in underlined.

**Fig 9:**
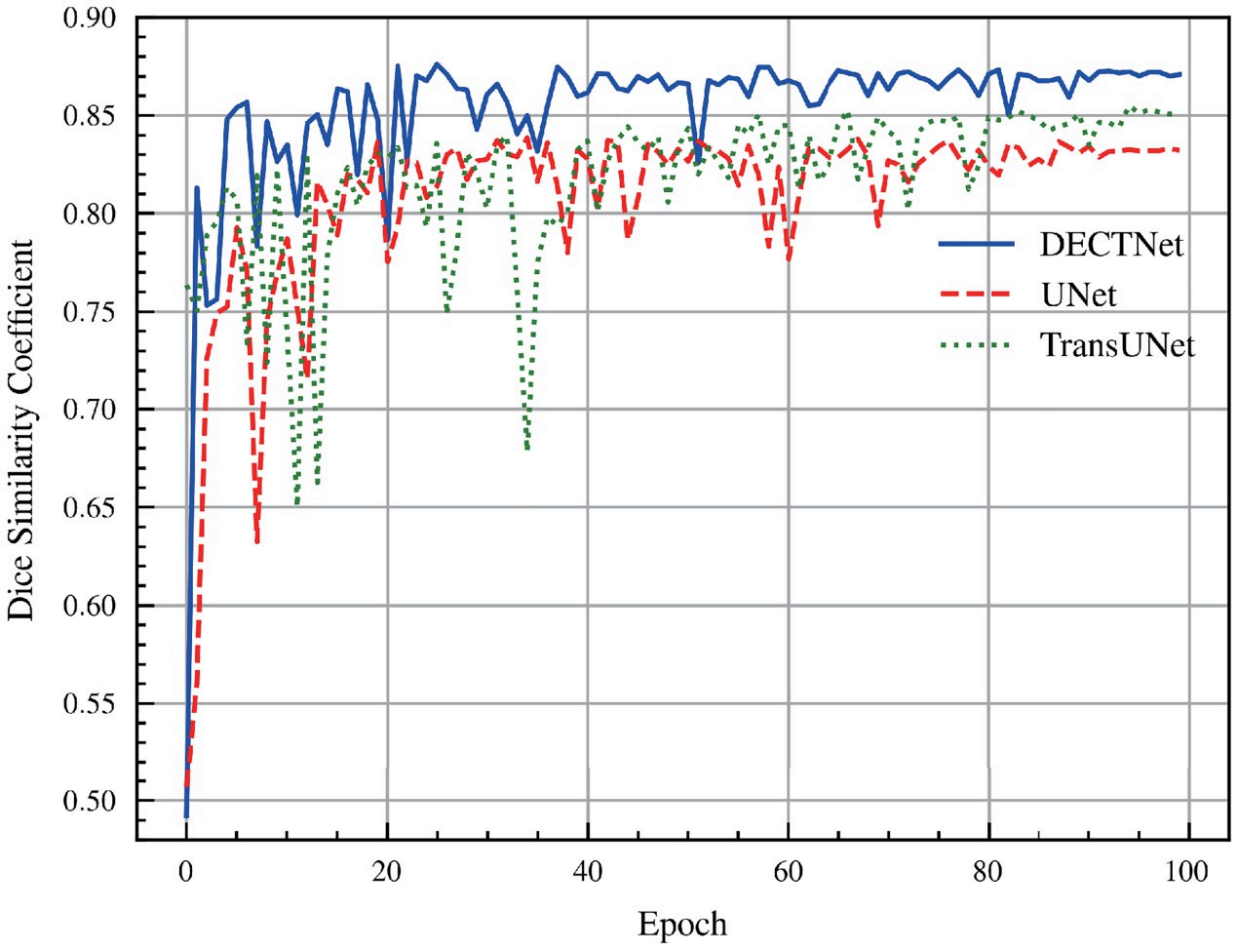
Comparison of Dice score among different methods on the validation dataset in the Covid-19 lesion segmentation task during the training process.

The components of Fig 8 are familiar to Fig 6. Compared with the masks from other segmentation methods, our model obtains more accurate segmentation results on some complicated boundary regions, and the segmentation masks closely match the shape and location of the infected region delineated by the ground truth. Besides, Fig 9 is also similar to Fig 7. From these curves, we observe that the Dice score of DECTNet consistently outperforms other models, which proves its better segmentation capability. However, compared with Fig 7, these curves in Fig 9 have large fluctuations, which the different distribution between different datasets may cause.

### Polyp segmentation

Further, we validate the segmentation performance of the proposed method in the polyp segmentation task. As described in Section **Experiments**, this segmentation task contains five different polyp segmentation datasets. In order to explicitly represent the generalization performance of different models, the evaluation metrics of different models on each polyp segmentation dataset are displayed in Table 3. For the convenience of presentation, we adopt Dice and Jaccard as the evaluation metrics. Table 3 shows that DECTNet achieves the highest mean Dice and Jaccard in the four datasets except for the ETIS dataset, which also achieves the suboptimal result. It proves that in comparison with other models, our DECTNet has improved generalization performance in the polyp segmentation task.

**Table 3:** Result of comparisons with other methods in polyp segmentation task.

	Metrics									
	Kvasir		ClinicDB		ColonDB		EndoScene		ETIS	
Method	Dice(%)	Jaccard(%)	Dice(%)	Jaccard(%)	Dice(%)	Jaccard(%)	Dice(%)	Jaccard(%)	Dice(%)	Jaccard(%)
UNet	84.21(0.46)	73.07(0.27)	88.99(0.53)	80.51(0.20)	65.87(0.68)	53.07(0.35)	79.51(0.49)	67.78(0.48)	50.42(0.85)	37.38(1.35)
UNet++	78.03(0.52)	64.74(0.26)	86.68(0.49)	77.32(0.49)	58.88(0.31)	45.26(0.42)	75.41(0.88)	61.97(0.39)	48.91(0.58)	36.69(0.86)
AttenUNet	84.62(0.60)	73.69(0.56)	88.72(0.29)	80.16(0.42)	67.27(0.43)	54.01(0.42)	78.06(0.15)	66.05(0.72)	50.93(0.68)	38.42(1.03)
DeepLabV3+	78.53(0.45)	65.36(0.90)	88.27(0.51)	79.47(0.30)	56.24(0.48)	42.09(0.69)	71.72(0.28)	59.55(0.53)	41.53(0.57)	29.27(1.24)
CENet	84.79(0.73)	74.05(0.69)	91.32(0.19)	84.12(0.16)	76.17(0.35)	65.19(0.56)	86.56(0.44)	76.67(0.35)	69.59(0.21)	55.25(0.55)
TransUNet	84.37(0.12)	73.73(0.25)	89.60(0.25)	81.63(0.43)	69.97(0.55)	57.41(0.61)	80.60(0.63)	68.85(0.59)	56.54(0.76)	44.64(1.01)
TransFuse	82.81(0.22)	71.13(0.31)	89.86(0.29)	81.73(0.56)	75.65(0.72)	63.11(0.86)	85.03(0.34)	74.83(0.41)	**70.47**(0.21)	**56.15**(0.41)
DECTNet	**87.56**(0.19)	**78.90**(0.32)	**91.73**(0.15)	**84.81**(0.29)	**77.92**(0.71)	**67.97**(0.51)	**86.95**(0.18)	**77.09**(0.34)	69.70(0.16)	55.68(0.33)

The values are described as Mean(Standard deviation). Best results are in bold and suboptimal results are in underlined.

**Fig 10:**
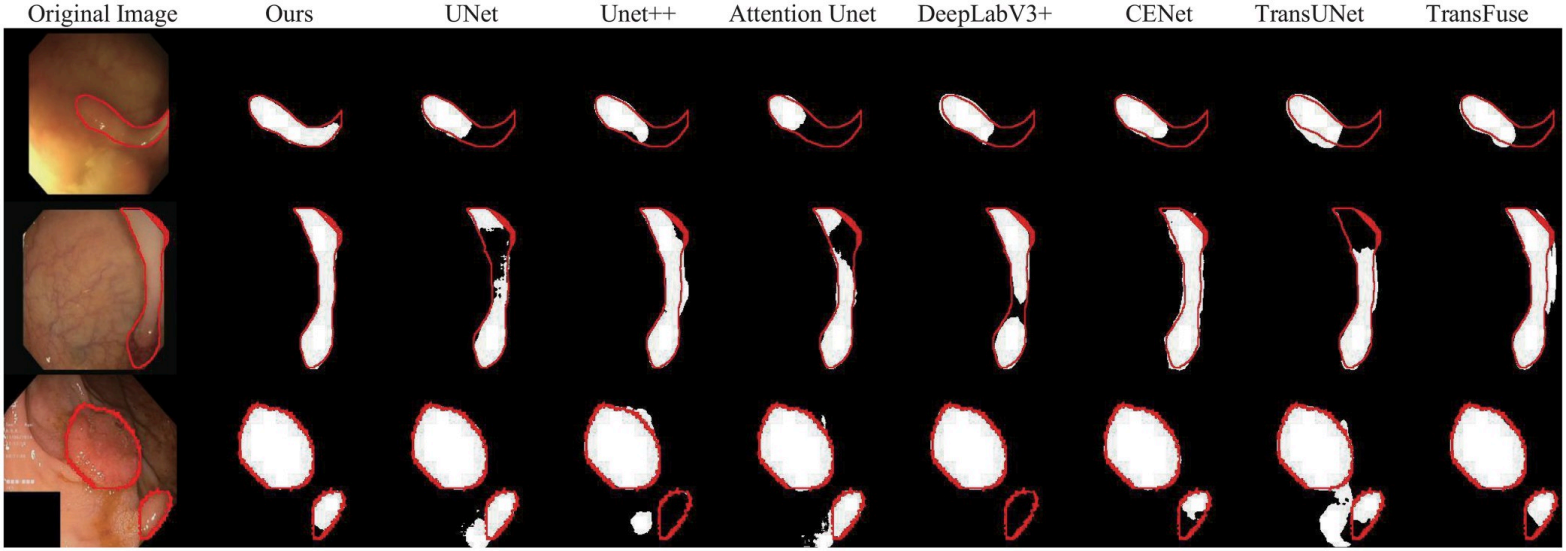
Visual comparison examples with other approaches in the polyp segmentation. The red contour refers to the ground truth, and different segmentation masks are produced by different methods.

**Fig 11:**
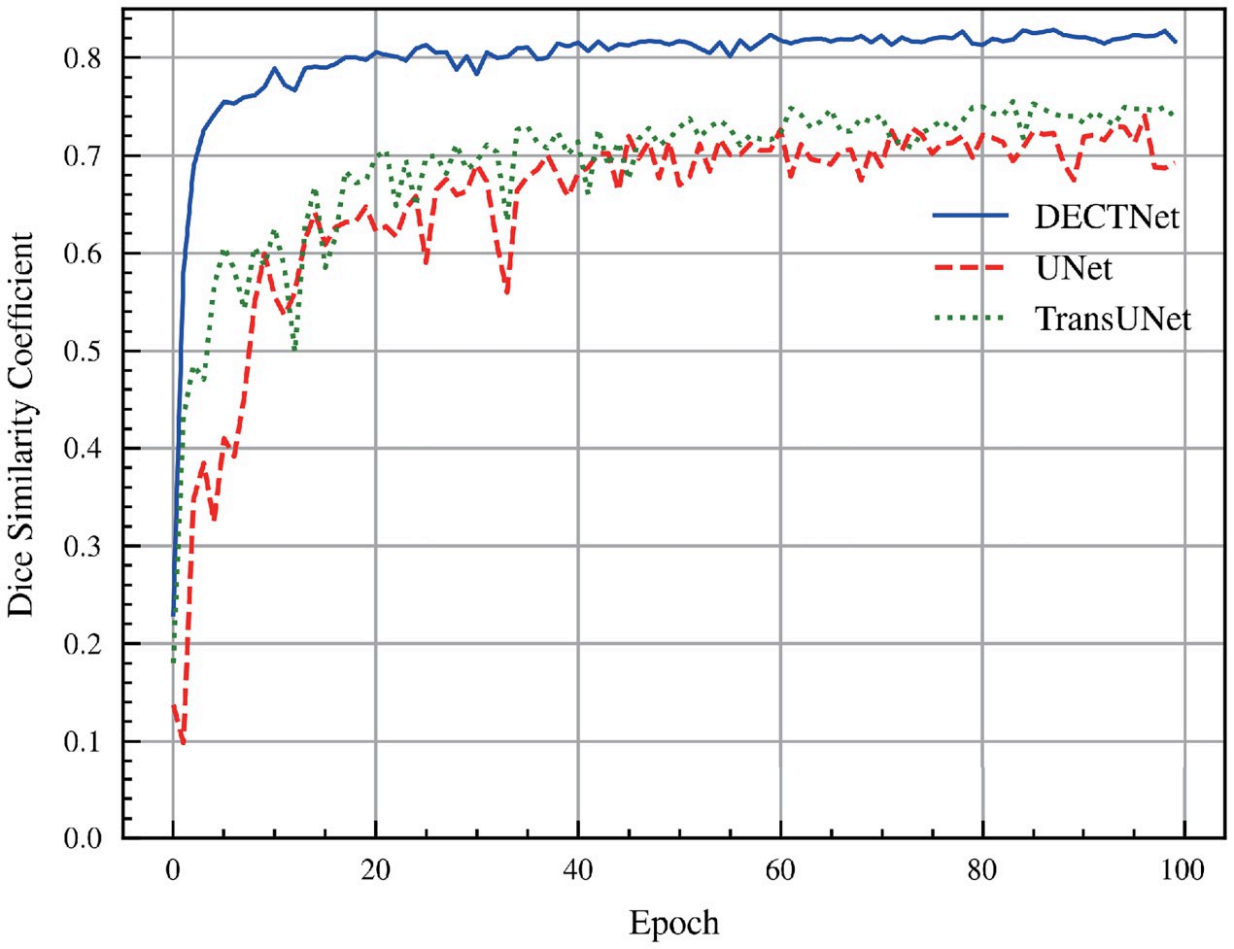
Comparison of Dice score among different methods on the validation dataset in the polyp segmentation task during the training process.

Fig 10 visualizes some segmentation masks different models produce. Compared to other counterparts, our method outlines the lesion regions more accurately and eliminates the background noise. Even in complicated samples, our model still generates a precise segmentation mask. In addition, Fig 11 shows the Dice score curves of different models on the polyp segmentation validation dataset. It can be seen that the Dice curve resulting from DECTNet has a considerable advantage compared with the other two models, which proves our proposed model has better generalization capability in the polyp segmentation task.

### Cardiac segmentation

We finally perform the comparison experiment of the DECTNet and other segmentation models in the cardiac segmentation task. Unlike the previous segmentation task based on binary classification, cardiac segmentation is a multi-class segmentation task, including the left ventricle, right ventricle, and myocardium. Table 4 gives the quantitative comparison results of different categories in the cardiac segmentation task. Following [59], the Dice, Jaccard, HD, and ASD are adopted as the segmentation performance metrics in this task, where HD and ASD are measured in pixels. And in Table 4, RV, MYO, and LV represent the right ventricle, myocardium, and left ventricle, while *p* refers to pixel. Table 4 indicated that our model achieved the best results in all evaluation metrics of LV segmentation and obtained the highest Dice, Jaccard, and comparable surface-related performance in RV and MYO segmentation compared with other methods. It demonstrated that our proposed DECTNet is effective for the cardiac segmentation task.

**Table 4:** Result of comparisons with other methods in cardiac segmentation task.

	Metrics											
	RV				MYO				LV			
Method	Dice(%)	Jaccard(%)	HD(*p*)	ASD(*p*)	Dice(%)	Jaccard(%)	HD(*p*)	ASD(*p*)	Dice(%)	Jaccard(%)	HD(*p*)	ASD(*p*)
UNet	92.98(0.23)	87.08(0.36)	4.82(0.27)	0.29(0.05)	85.58(0.22)	74.98(0.35)	6.39(2.02)	0.32(0.09)	88.42(0.29)	79.72(0.40)	9.14(0.64)	0.33(0.05)
UNet++	92.72(0.26)	86.71(0.41)	5.75(0.78)	0.30(0.3)	84.78(0.24)	73.87(0.42)	7.51(1.42)	0.47(0.19)	88.59(0.26)	79.97(0.44)	8.15(1.06)	0.33(0.07)
AttnUNet	92.04(0.30)	85.56(0.48)	4.47(0.24)	0.33(0.02)	83.87(0.19)	72.44(0.26)	7.23(1.23)	0.53(0.10)	87.31(0.19)	78.03(0.22)	8.92(1.05)	0.42(0.06)
DeepLabV3+	91.94(0.44)	85.43(0.59)	6.83(0.76)	0.35(0.03)	83.72(0.51)	72.22(0.44)	9.68(1.29)	0.43(0.08)	85.98(0.49)	76.04(0.61)	9.81(1.36)	0.45(0.11)
CENet	93.06(0.26)	87.23(0.57)	4.04(0.78)	0.22(0.01)	84.66(0.58)	73.58(0.42)	5.27(0.40)	0.32(0.02)	88.55(0.61)	79.68(0.77)	7.01(0.85)	0.30(0.02)
TransUNet	93.15(0.19)	88.16(0.48)	4.55(0.70)	0.22(0.01)	86.58(0.55)	76.50(0.46)	6.52(0.89)	0.36(0.03)	89.52(0.45)	81.57(0.55)	6.16(0.57)	0.28(0.02)
TransFuse	92.76(0.31)	86.77(0.45)	**3.54**(0.15)	**0.19**(0.01)	84.41(0.56)	73.17(0.65)	**4.62**(0.30)	**0.31**(0.02)	88.01(0.66)	79.13(0.71)	5.91(0.32)	0.30(0.02)
DECTNet	**93.80**(0.20)	**88.65**(0.31)	3.83(0.21)	0.29(0.02)	**87.12**(0.45)	**77.51**(0.30)	5.14(0.52)	0.37(0.03)	**90.17**(0.15)	**82.57**(0.20)	**5.47**(0.35)	**0.22**(0.01)

The values are described as Mean(Standard deviation). Best results are in bold and suboptimal results are in underlined.

**Fig 12:**
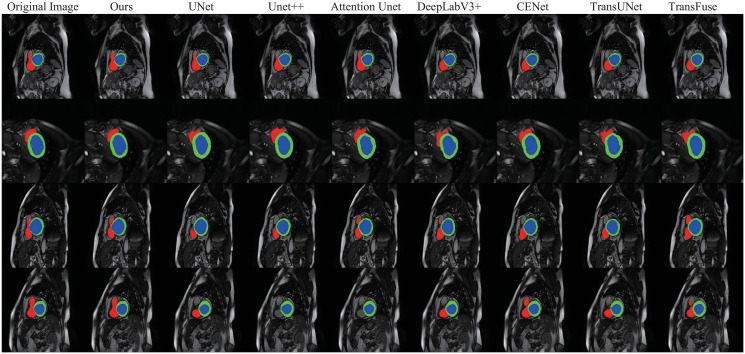
Visual comparison examples with other approaches in the cardiac segmentation. The red, green, and blue portions refer to the right ventricle, the myocardium, and the left ventricle, respectively. Different segmentation masks are produced by different methods, where the masks in “Original Image” refer to the ground truth.

Fig 12 also gives a few samples for visual comparison. Different from visual comparison examples in preceding segmentation tasks, in Fig 12, the red, green, and blue regions respectively correspond to the segmentation masks of the right ventricle, myocardium, and left ventricle, where the segmentation masks in the “Original Image” are labeled with corresponding ground truth. Compared with other segmentation masks, the superiority of segmentation masks resulting from our method is in the region of the right ventricle(red). It is observed from the visual samples that for the left ventricle(blue) and myocardium(green) regions, the segmentation masks produced by different methods have little difference concerning the ground truth. But for the cases of the right ventricle(red), our segmentation masks have tremendous advantages compared with other masks. Our method enables us to distinguish the target region more accurately.

**Fig 13:**
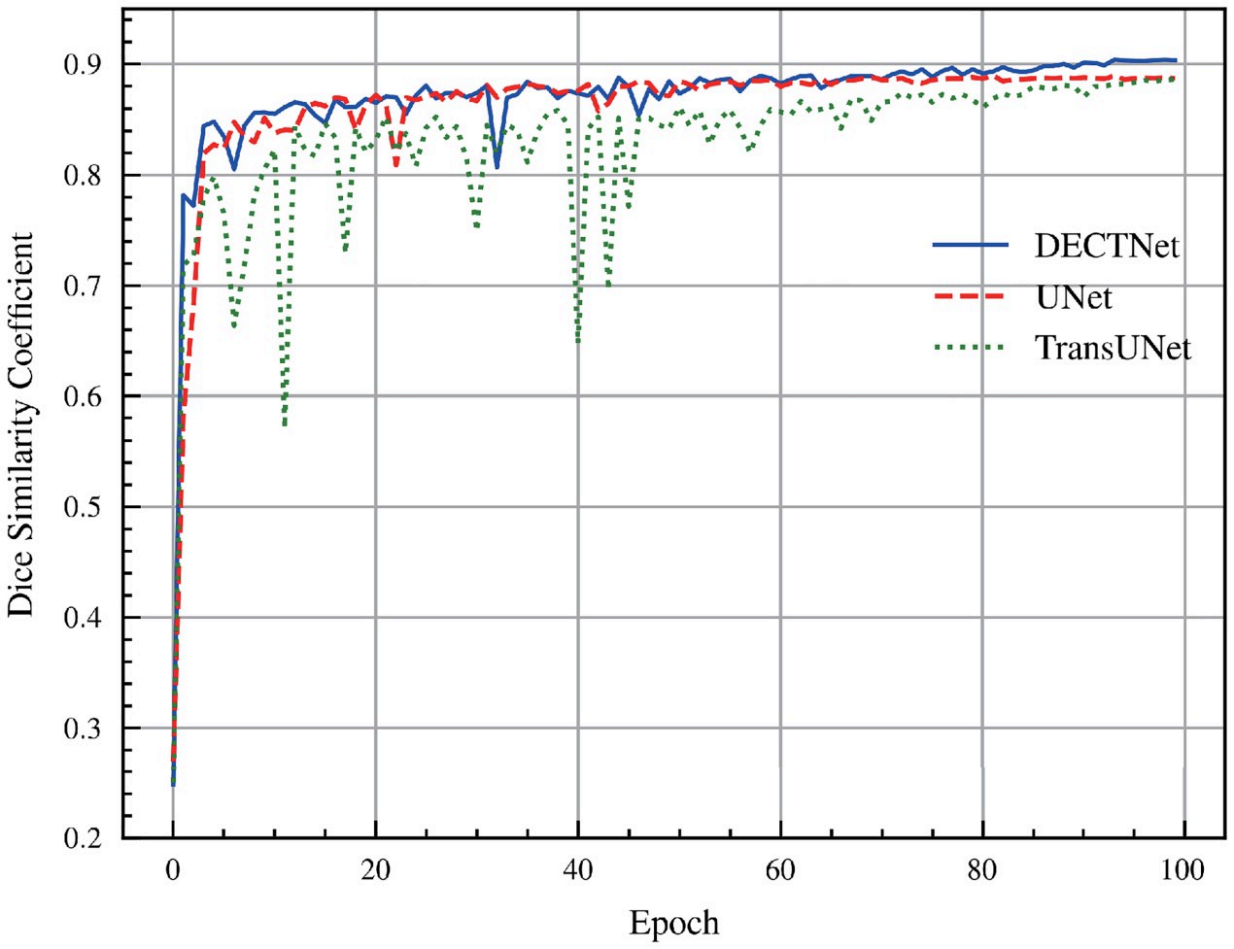
Comparison of Dice score among different methods on the validation dataset in the cardiac segmentation task during the training process.

As shown in Fig 13, we also validate the Dice scores of different segmentation models on the validation dataset during training. Different from the previous three segmentation tasks, we describe the mean Dice scores of the three categories(RV, MYO, LV) in Fig 13. It can be shown that the Dice score of our model on the validation dataset still slightly outperforms other segmentation models.

### Ablation study

**Fig 14:**
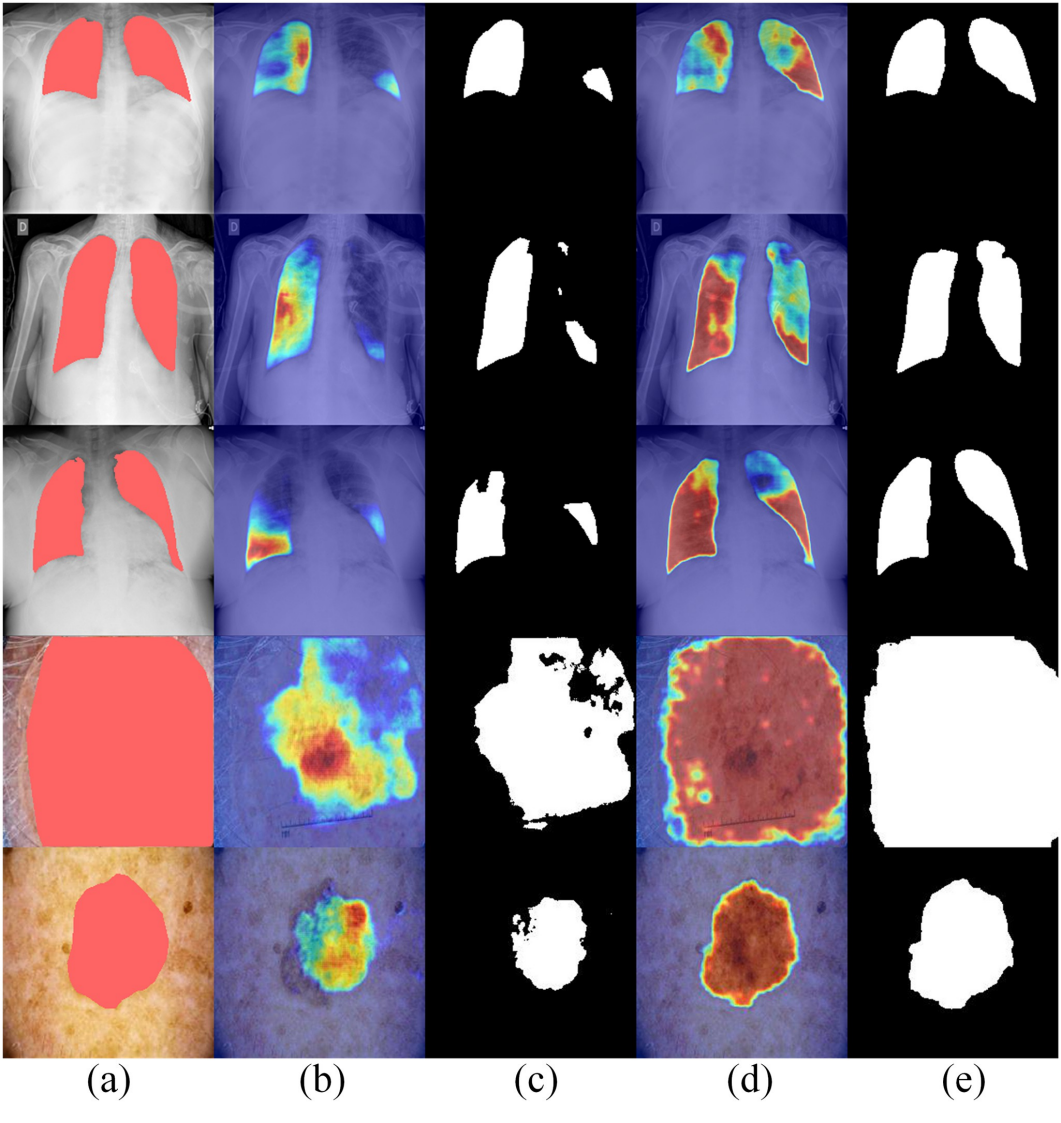
Visual comparison samples in Ablation study. (a) refers to the original image and the corresponding ground truth, (b) and (c) refer to the heat map and segmentation mask produced by “C-encoder + Sim decoder”, and (d) and (e) to the heat map and segmentation mask produced by the DECTNet.

In order to investigate the effectiveness of the proposed model and its components, we evaluate the DECTNet on skin lesion segmentation task and Covid-19 lesion segmentation task for ablation study. In the ablation experiments, we primarily validate the effects of four modules: the encoder module based on Convolution architecture, the encoder module based on Transformer architecture, the decoder module with feature integration and selection module, and the deep supervision module. Tables 5 and 6, and Fig 14 respectively show the quantitative results and visual comparisons of the ablation experiments in two segmentation tasks. In Tables 5 and 6, the “C-encoder + Sim decoder” refers to the combination of the CNN-based encoder and the simple decoder based on UNet. The “T-encoder” refers to the encoder based on Swin Transformer architecture. Different from the simple decoder in previous structures, the decoder in the “Dual-encoder + Sim decoder” architecture adjusts the scales of feature maps from different encoders with additional convolution layers. The “w/o DeepSup” structure indicates the DECTNet without the deep supervision module. Note that the segmentation models containing the swim transformer structure are trained from scratch without pre-trained parameters.

**Table 5:** Quantitative result of ablation study in skin lesion segmentation task.

	Metrics					
Method	Dice(%)	Jaccard(%)	Accuracy(%)	Sensitivity(%)	Precision(%)	Specificity(%)
UNet	82.58(0.51)	73.40(0.61)	92.32(0.05)	81.33(0.30)	90.31(0.18)	96.44(0.19)
C-encoder + Sim decoder	83.57(0.28)	73.53(0.37)	93.81(0.15)	84.68(0.34)	88.44(0.22)	97.50(0.20)
T-encoder + Sim decoder	74.23(1.16)	64.76(1.45)	89.90(0.29)	67.76(0.88)	91.53(0.44)	94.68(0.39)
Dual-encoder + Sim decoder	85.33(0.41)	75.24(0.22)	**94.98**(0.25)	83.48(0.81)	88.83(0.60)	97.49(0.15)
w/o DeepSup	86.05(0.42)	77.21(0.55)	94.79(0.18)	**84.76**(0.53)	89.44(0.59)	**97.61**(0.10)
DECTNet	**86.36**(0.38)	**78.38**(0.49)	94.91(0.13)	84.46(0.31)	**92.40**(0.36)	97.12(0.12)

The values are described as Mean(Standard deviation). Best results are in bold and suboptimal results are in underlined.

**Table 6:** Quantitative result of ablation study in Covid-19 lesion segmentation task.

	Metrics					
Method	Dice(%)	Jaccard(%)	Accuracy(%)	Sensitivity(%)	Precision(%)	Specificity(%)
UNet	83.87(0.31)	72.92(0.67)	96.58(0.45)	87.04(0.86)	81.31(0.89)	97.65(0.12)
C-encoder + Sim decoder	84.15(0.48)	73.79(0.37)	97.11(0.21)	85.82(0.77)	85.18(0.70)	98.22(0.25)
T-encoder + Sim decode	73.27(1.56)	59.27(1.71)	95.44(0.60)	61.81(2.11)	83.55(0.66)	94.45(0.75)
Dual-encoder + Sim decoder	85.93(0.65)	75.84(0.50)	97.19(0.35)	87.89(0.69)	**89.55**(0.17)	**98.88**(0.13)
w/o DeepSup	86.29(0.71)	76.42(0.49)	97.20(0.44)	**89.01**(0.77)	86.26(0.44)	98.39(0.27)
DECTNet	**87.99**(0.37)	**78.96**(0.21)	**97.50**(0.46)	89.00(0.50)	87.27(0.63)	98.51(0.28)

The values are described as Mean(Standard deviation). Best results are in bold and suboptimal results are in underlined.

From Tables 5 and 6, it is observed that compared to the segmentation performance of UNet, the evaluation metrics acquired by the “C-encoder + Sim decoder” model are enhanced, which shows the capability of the designed module in the C-encoder structure. The segmentation performance of the “T-encoder + Sim decoder” model is significantly degraded, probably due to insufficient training samples to make the Swin Transformer structure effective alone. Nevertheless, the “Dual-encoder + Sim decoder” structure achieves superior segmentation performance than the “C-encoder + Sim decoder” and “T-encoder + Sim decoder” structures, which demonstrates that the Swin Transformer structure could capture the effective representations that are difficult to obtain with convolutional structures. In addition, the enhanced capability of the “w/o DeepSup” and DECTNet structures demonstrate the benefits of the feature integration and selection module and the deep supervision module, respectively.

Increasing studies have been developed to improve the interpretability of segmentation methods through CAM-like approaches [60, 61] to enhance the confidence of prediction results of deep learning models. Following [61], we create the heat maps based on grad-CAM [60] to visualize and interpret the segmentation results of the proposed DECTNet.

As shown in Fig 14, we take the heatmaps and segmentation masks for comparison. The (a) refers to the samples and the corresponding labels from two segmentation datasets, where the red regions represent labels. The (b) and (c) refer to the heatmaps and the corresponding segmentation masks resulting from the “C-encoder + Sim decoder” structure. The resulting heatmaps and segmentation masks produced by the DECTNet are represented by (d) and (e). As can be obtained from Fig 14, compared with (b) and (c), (d) and (e) are better suited to the corresponding label. It demonstrates that the designed modules for the DECTNet can precisely and steadily extract targets for medical image segmentation tasks.

## Discussion

To achieve better accurate and robust performance in the medical image segmentation domain, we propose the DECTNet achieve this purpose. The DECTNet is a dual-encoder method based on Convolution and Swin Transformer architecture. To efficiently acquire local context information from images, we design the convolution encoder stage consisting of a Dense Connection block and a CBAM block, where the Dense Connection block delivers abundant semantic information, and the CBAM block utilizes spatial and channel attention masks to enhance semantic features that are beneficial for specific segmentation tasks. Furthermore, we employ depth-separable convolution instead of traditional convolution in the convolution encoder to reduce the model parameters. To better capture the global context dependence of medical images, we design the Swin Transformer encoder in the proposed DECTNet. Moreover, we incorporate the STP blocks in the Swin Transformer encoder for the preliminary image processing to make the Swin Transformer structure suitable for semantic segmentation tasks. Since different encoders yield different scale features, we design a feature fusion decoder to integrate and select features considered significant for the segmentation tasks. In addition, to accelerate the convergence and improve the segmentation performance of the proposed method, we add a simple but effective deep supervision module to supervise the decoder stage outputs.

To perform the performance of the proposed DECTNet, we evaluate the method on four different medical image segmentation tasks to demonstrate its effectiveness and robustness. The comparison experimental results corresponding to Tables 1–4 show that the DECTNet achieves state-of-the-art results than other segmentation models. Figs 6, 8, 10 and 12 show that our model has superior segmentation accuracy and generalization performance compared to other segmentation models. To further prove the efficacy of each component of the proposed DECTNet, we perform ablation studies on the skin lesion segmentation task and the Covid-19 lesion segmentation task. The corresponding figures and tables of the ablation experiments show that the dual encoder structure with convolution and Swin Transformer architecture improves the model’s performance compared to a single structure encoder. Moreover, it is also seen that the feature fusion decoder and the deep supervision module also contribute to improving the performance of the proposed method. In conclusion, compared with other state-of-the-art models, DECTNet is effective in improving the accuracy of image segmentation and obtaining a favorable generalization of the segmentation method.

Compared to 2D segmentation networks such as DECTNet, 3D models allow direct processing of 3D data, which enables the segmentation model to capture volume information and efficiently understand more comprehensive spatial context representation. However, due to the parameter redundancy in the dual encoder structure of the 2D DECTNet, we need to reoptimize the model parameters to develop a robust and generalizable 3D DECTNet method.

In contrast to the single-encoder segmentation network, the dual-encoder structure of DECTNet increases the model complexity and requires additional annotation data to mitigate the overfitting risks. Besides that, this complexity expands the number of hyperparameters, making it more difficult and time-consuming to find the best hyperparameter configurations. Future enhancements can be approached in two ways. On the one hand, leveraging transfer learning, complex models are pre-trained on sufficiently large datasets and establish a more effective framework, which allows complex models to obtain more robust generic visual features and diminishes the possibility of overfitting. On the other hand, based on the Transformer architecture, the model structures can be refined by developing a less parameterized yet more efficient global feature extraction module, which aims to enhance the efficiency and accuracy of the segmentation backbone.

## Conclusion

In this paper, we propose a novel model with a dual encoder structure named DECTNet, for medical image segmentation. We use the convolution encoder and Swin Transformer encoder to extract local and global context information to obtain the significant hierarchical representation from medical images. Then, we design a feature fusion decoder to integrate and select the representation acquired from the convolution and Swin Transformer encoders. Further, We employ a deep supervision module to supervise the multi-scale features in the decoder stages. Our method demonstrates significant advantages through the experiments compared to the state-of-the-art methods on four public segmentation tasks.

## Supporting information

S1 Fig Some visual comparison samples in skin lesion segmentation task. The red portion indicates segmentation maps generated by different models and corresponding ground truth. (i) refer to the original images and the corresponding labels, (ii) and (iii) refer to the Grad-CAM heatmaps and corresponding segmentation maps generated by DECTNet, (iv) and (v) are generated by CENet, (vi) and (vii) are generated by TransUNet, and (viii) and (ix) are generated by UNet.
(TIF)

S2 Fig Some visual comparison samples in Covid-19 lesion segmentation task. The red portion indicates segmentation maps generated by different models and corresponding ground truth. (i) refer to the original images and the corresponding labels, (ii) and (iii) refer to the Grad-CAM heatmaps and corresponding segmentation maps generated by DECTNet, (iv) and (v) are generated by CENet, (vi) and (vii) are generated by TransUNet, and (viii) and (ix) are generated by UNet.
(TIF)

S3 Fig Some visual comparison samples in polyp segmentation task. The red portion indicates segmentation maps generated by different models and corresponding ground truth. (i) refer to the original images and the corresponding labels, (ii) and (iii) refer to the Grad-CAM heatmaps and corresponding segmentation maps generated by DECTNet, (iv) and (v) are generated by CENet, (vi) and (vii) are generated by TransUNet, and (viii) and (ix) are generated by UNet.
(TIF)

S4 Fig Some visual comparison samples in cardiac segmentation task. The red portion indicates segmentation maps generated by different models and corresponding ground truth. (i) refer to the original images and the corresponding labels, (ii) and (iii) refer to the Grad-CAM heatmaps and corresponding segmentation maps generated by DECTNet, (iv) and (v) are generated by CENet, (vi) and (vii) are generated by TransUNet, and (viii) and (ix) are generated by UNet.
(TIF)
