# Supplementary figures and images for: DECTNet: Dual Encoder Network combined convolution and Transformer architecture for medical image segmentation
Source: PLoS One. 2024 Apr 4;19(4):e0301019. doi: 10.1371/journal.pone.0301019 (PMC10994332; doi:10.1371/journal.pone.0301019)

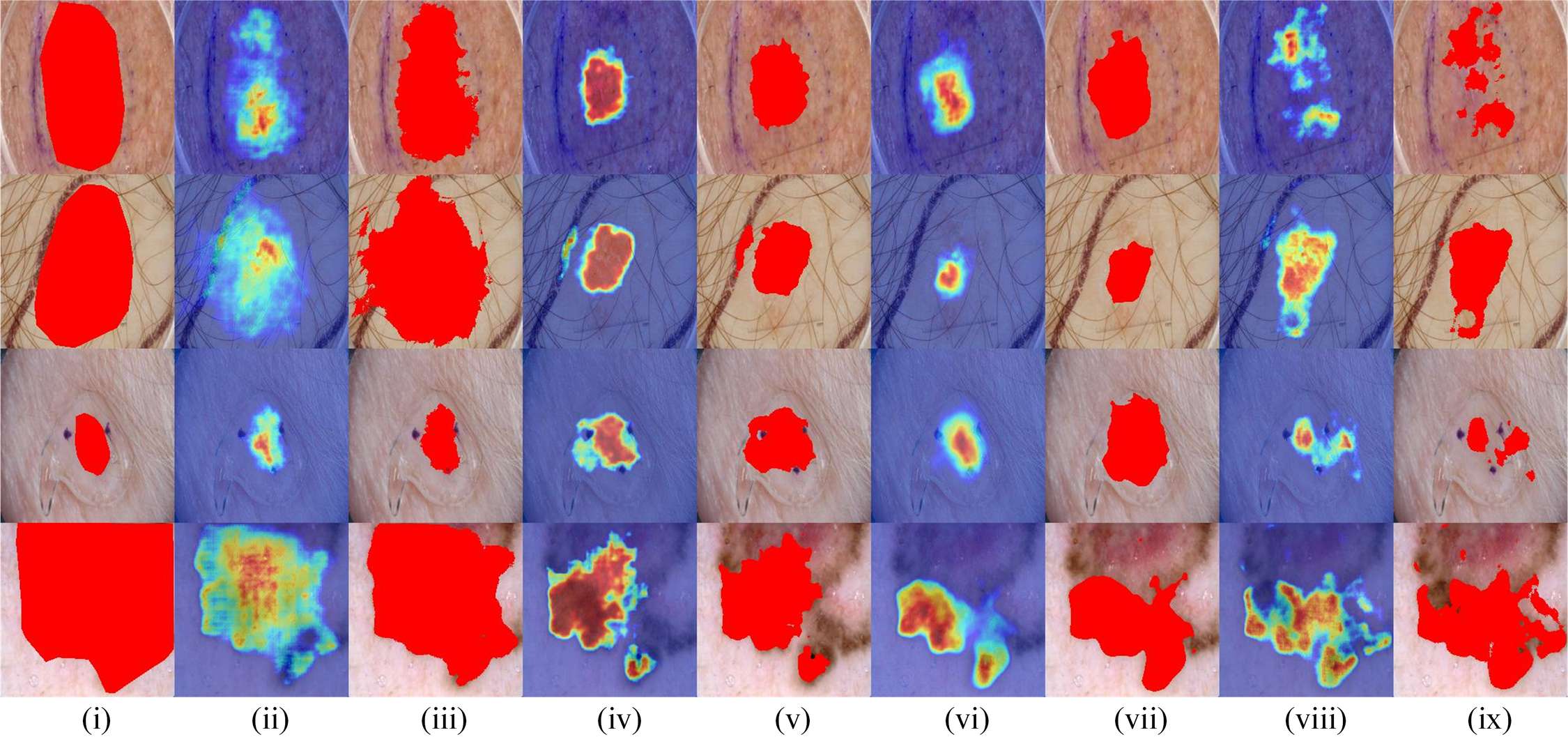

Supplement: S1 Fig — The red portion indicates segmentation maps generated by different models and corresponding ground truth. (i) refer to the original images and the corresponding labels, (ii) and (iii) refer to the Grad-CAM heatmaps and corresponding segmentation maps generated by DECTNet, (iv) and (v) are generated by CENet, (vi) and (vii) are generated by TransUNet, and (viii) and (ix) are generated by UNet. (TIF) [file pone.0301019.s001.tif]

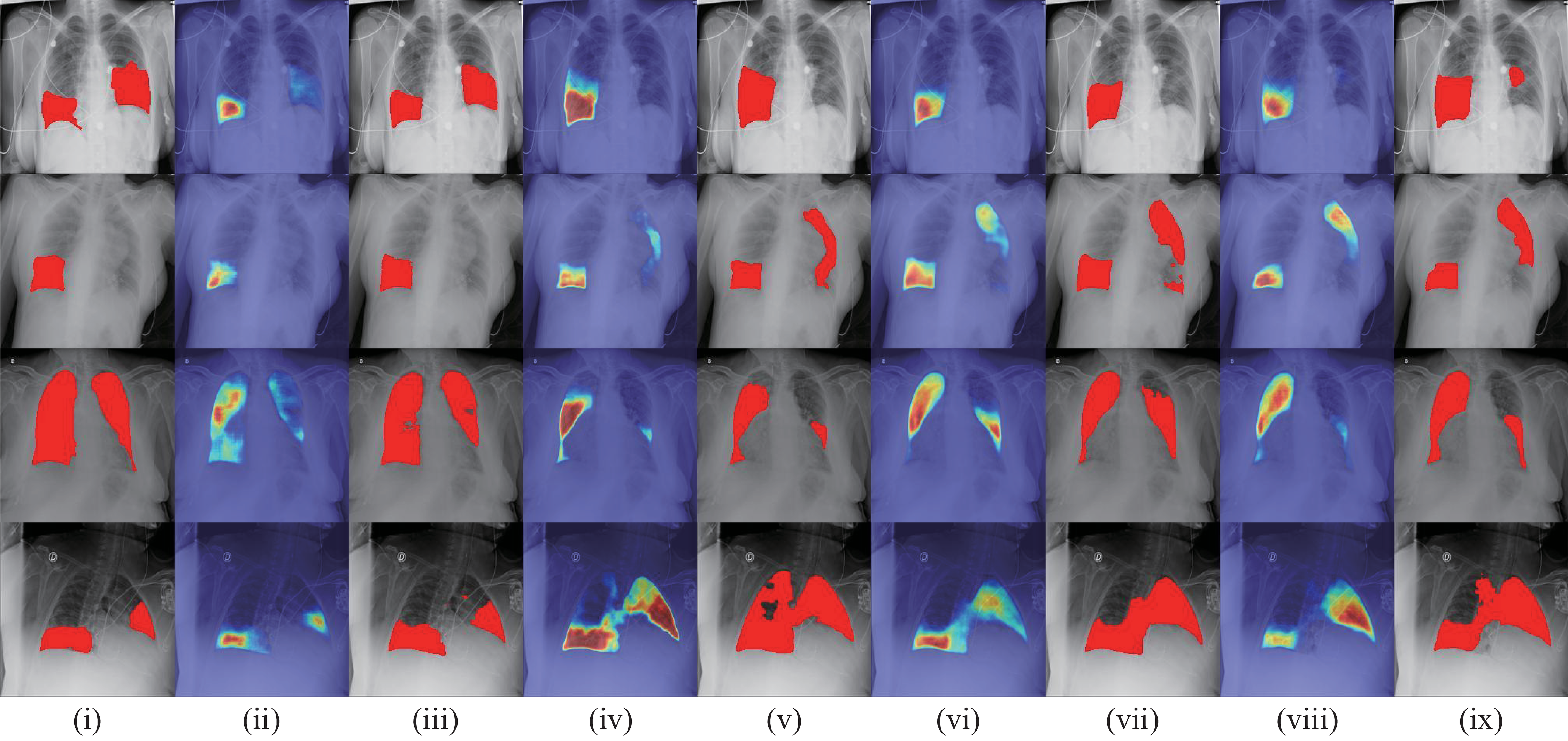

Supplement: S2 Fig — The red portion indicates segmentation maps generated by different models and corresponding ground truth. (i) refer to the original images and the corresponding labels, (ii) and (iii) refer to the Grad-CAM heatmaps and corresponding segmentation maps generated by DECTNet, (iv) and (v) are generated by CENet, (vi) and (vii) are generated by TransUNet, and (viii) and (ix) are generated by UNet. (TIF) [file pone.0301019.s002.tif]

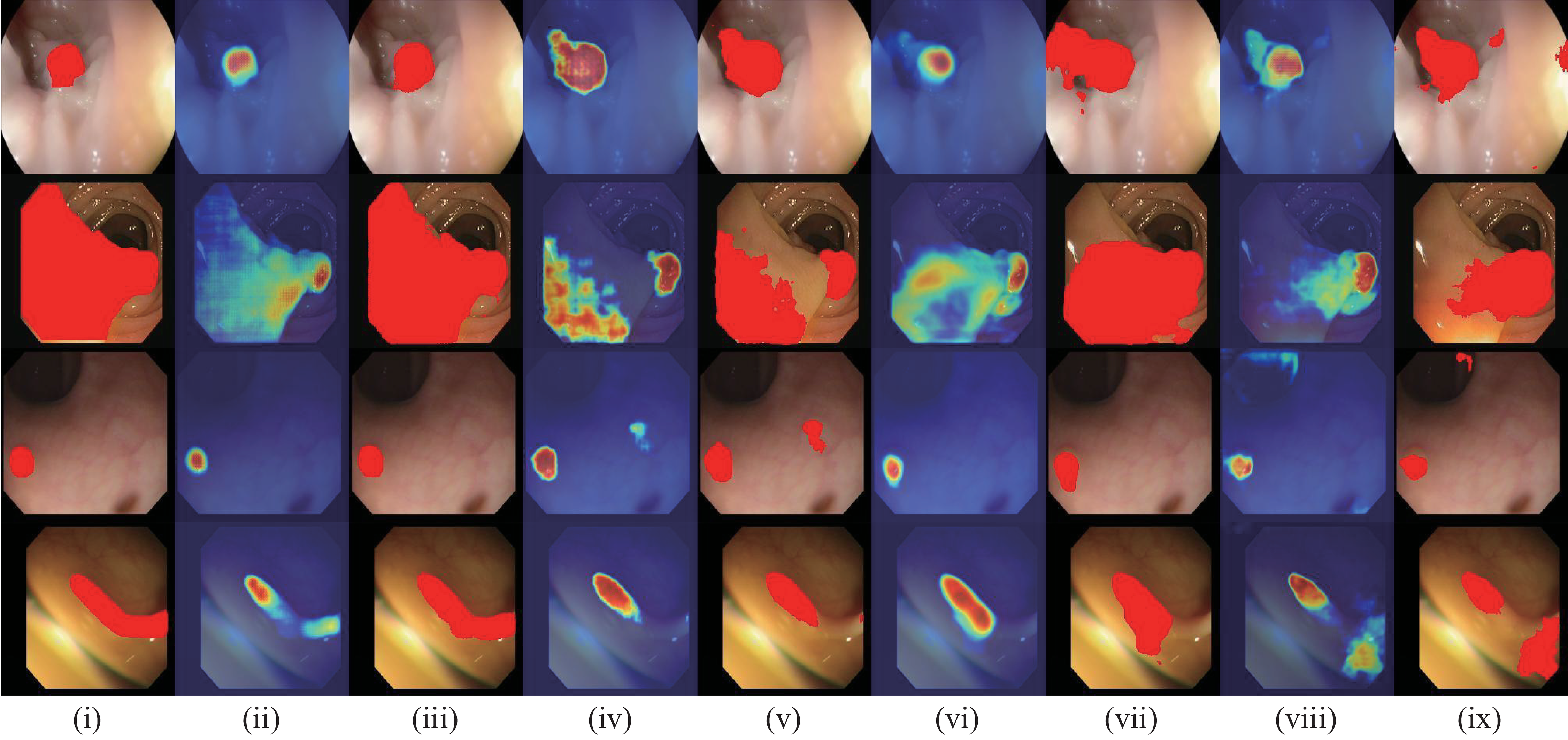

Supplement: S3 Fig — The red portion indicates segmentation maps generated by different models and corresponding ground truth. (i) refer to the original images and the corresponding labels, (ii) and (iii) refer to the Grad-CAM heatmaps and corresponding segmentation maps generated by DECTNet, (iv) and (v) are generated by CENet, (vi) and (vii) are generated by TransUNet, and (viii) and (ix) are generated by UNet. (TIF) [file pone.0301019.s003.tif]

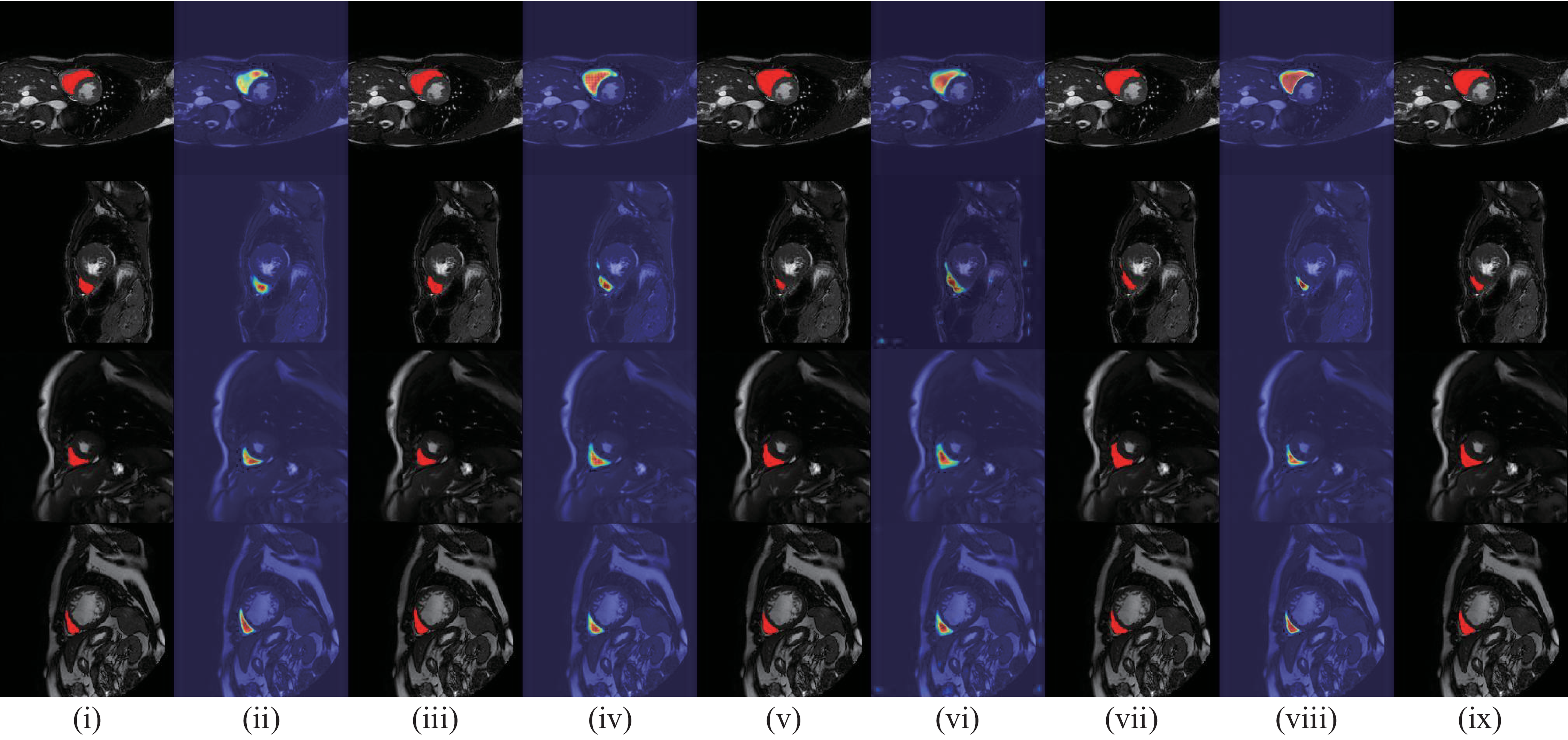

Supplement: S4 Fig — The red portion indicates segmentation maps generated by different models and corresponding ground truth. (i) refer to the original images and the corresponding labels, (ii) and (iii) refer to the Grad-CAM heatmaps and corresponding segmentation maps generated by DECTNet, (iv) and (v) are generated by CENet, (vi) and (vii) are generated by TransUNet, and (viii) and (ix) are generated by UNet. (TIF) [file pone.0301019.s004.tif]
